# Ischemic cardiac stromal fibroblast-derived protein mediators in the infarcted myocardium and transcriptomic profiling at single cell resolution

**DOI:** 10.1007/s10142-024-01457-1

**Published:** 2024-09-20

**Authors:** Ed Cha, Sung Ho Hong, Taj Rai, Vy La, Pranav Madabhushi, Darren Teramoto, Cameron Fung, Pauline Cheng, Yu Chen, Angelo Keklikian, Jeffrey Liu, William Fang, Finosh G. Thankam

**Affiliations:** 1 Department of Translational Research, College of Osteopathic Medicine of the Pacific, Western University of Health Sciences, 309 E. Second Street, Pomona, CA 91766-1854 USA; 2 Department of Biology, University of California San Diego, La Jolla, CA 92093 USA; 3 Molecular Instrumentation Center, University of California-Los Angeles, Los Angeles, CA 90095 USA

**Keywords:** Myocardial infarction, Cardiac stromal fibroblasts, Sub-phenotypes, Infarct zone, Ischemia and reperfusion

## Abstract

This article focuses on screening the major secreted proteins by the ischemia-challenged cardiac stromal fibroblasts (CF), the assessment of their expression status and functional role in the post-ischemic left ventricle (LV) and in the ischemia-challenged CF culture and to phenotype CF at single cell resolution based on the positivity of the identified mediators. The expression level of CRSP2, HSP27, IL-8, Cofilin-1, and HSP90 in the LV tissues following coronary artery bypass graft (CABG) and myocardial infarction (MI) and CF cells followed the screening profile derived from the MS/MS findings. The histology data unveiled ECM disorganization, inflammation and fibrosis reflecting the ischemic pathology. CRSP2, HSP27, and HSP90 were significantly upregulated in the LV-CABG tissues with a concomitant reduction ion LV-MI whereas Cofilin-1, IL8, Nrf2, and Troponin I were downregulated in LV-CABG and increased in LV-MI. Similar trends were exhibited by ischemic CF. Single cell transcriptomics revealed multiple sub-phenotypes of CF based on their respective upregulation of CRSP2, HSP27, IL-8, Cofilin-1, HSP90, Troponin I and Nrf2 unveiling pathological and pro-healing phenotypes. Further investigations regarding the underlying signaling mechanisms and validation of sub-populations would offer novel translational avenues for the management of cardiac diseases.

**Supplementary Information:**

The online version contains supplementary material available at 10.1007/s10142-024-01457-1.

## Introduction

Myocardial infarction (MI) resulting from coronary artery disease (CAD) accounts for about 21% of total deaths in the United States with an alarming increase of about 5% since 2012 (Ahmad and Anderson 2021). Around 25% of cardiomyocytes (depending on the severity of injury) from the left ventricle, accounting for ~ 1 billion cells, and the local coronary vessels in the myocardium are permanently damaged following the ischemic episodes associated with MI (Giacca 2020). Furthermore, the infarct zone (IZ) is the primary site of aggravated pathology caused by the persistent immune reactions subsequently resulting in left ventricular remodeling (Ma et al. 2019). The inherent cardiac repair mechanism following MI intensifies the fibrotic pathways leading to scar tissue at the IZ impairing the cardiac function (Thankam et al. 2022d). Conventional treatment strategies including percutaneous coronary intervention (PCI), coronary artery bypass graft (CABG), and pharmacotherapy such as diuretics, statins, thrombolytic drugs, angiotensin-converting enzyme (ACE) inhibitors and beta-blockers alleviate the symptoms; however, the cellular and molecular interplay at the IZ for compensating the massive loss of cardiomyocyte following ischemic insults are largely unknown (Cui et al. 2018). Unfortunately, the challenge of persistent ischemia at the site of anastomoses in post-CABG survivors significantly affects performance of the myocardium (Thankam et al. 2020).

Response among the major non-cardiomyocyte stromal cells including the CF, at the vicinity of IZ is crucial for eliciting the regenerative/pathological signaling and the communication among these cells via the secreted mediators operates the inflammatory/healing responses. Information regarding such secreted mediators by ischemia challenged CF are largely unknown; however, it is translationally relevant as CF proliferation attempts to replenish the cellular pool compensating the loss of cardiomyocytes (Burke et al. 2021) (Hall et al. 2021). Being the major non-cardiomyocyte cell type, ischemic insults alter the mediators secreted by the CF and the identification of the key mediators along with their expression status and functional role in ischemic left ventricular (LV) myocardium and CF would provide mechanical insights into the MI pathology. On this background, this article focuses on screening the major protein mediators secreted by the ischemia challenged CF cells, the assessment of their expression status and functional role in the post-ischemic LV and in the ischemia challenged CF culture and to phenotype CF at single cell resolution based on the upregulation of the identified mediators. The post-ischemic LVs used for this study were harvested from our previously reported translationally worthwhile swine models of CABG (Radwan et al. 2022) and acute MI (Thankam et al. 2022a).

## Methodology

### Swine model for CABG

The animal protocol for establishing the CABG model was approved by the Institutional Animal Care and Use Committee (IACUC) of Creighton University and the details of the CABG procedure are provided in our recent publication [10]. Briefly, four hyperlipidemic Yucatan micro-swine (*Sus scorfa*, Sinclair bioresources) which survived the CABG procedure were utilized in this study. CABG was performed by anastomosing the SEV (superficial epigastric vein) at left internal mammalian artery (LIMA) and diagonal branch of left anterior descending (LAD) artery as detailed in our previous publications (Radwan et al. 2022) (Thankam et al. 2020). The animals were sacrificed after 6 months and the ischemic myocardium surrounding the distal anastomosis (LV-CABG group) and the non-ischemic left ventricular (LV) tissue (CABG control) (LV-CABG-C) (*n* = 4) were harvested for further studies.

### Minimally invasive swine model for acute MI

The animal protocol for establishing the minimally invasive swine-MI model was approved by the Institutional Animal Care and Use Committee of Western University of Health Sciences, Pomona, California, USA. Four Yucatan mini swine (*Sus scorfa*, from Sinclair bioresources) were utilized for the study. MI was induced by angiography guided occlusion of coronary artery for 10–15 min using a balloon catheter following our recent report (Thankam et al. 2022c). The MI model simulated clinical MI as confirmed by the alterations in ECG, decreased ejection fraction, increased level of circulatory MI-biomarkers and pathological histomorphometry (Thankam et al. 2022c). The animals were sacrificed within a week following MI and the tissues at infarct zone (MI group) (LV-MI) was harvested for further studies. The myocardial tissues harvested from similar anatomical locations of mini pigs without MI served as control (LV-C) (Control group) (*n* = 4).

### Isolation, culture, and maintenance of CF

The post-sacrificed MI pigs and control pigs were utilized to isolate CF from left ventricular myocardium employing collagenase digestion according to our reported protocol (Thankam et al. 2019). The isolated CF were maintained in DMEM with 20% fetal bovine serum (FBS) (Cat# 30–2020, ATCC) under standard culture conditions (5% CO_2_, 37 °C, and antibiotics). The tissue for cell isolation was harvested from the same anatomical location in all animals. The CF were characterized based on the immunopositivity status of specific biomarkers as previously reported (Thankam et al. 2018a). The cells from passage 0–4 were used and ischemia was simulated in culture by treatment with ischemic buffer (118 mM NaCl, 24 mM Na_2_HCO_3_, 1 mM Na_2_HPO_4_, 2.5 mM CaCl_2_, 1.2 mM, MgCl_2_, 20 mM sodium lactate, 16 mM KCl, 10 mM 2-deoxyglucose and pH 6.2) (ISC group) for 2 h and reperfusion (ISC/R group) was simulated by replacing the complete media overnight following the ischemia challenge (Thankam and Agrawal 2021). The CF grown in normal DMEM were used as respective controls.

### Identification of CF-secreted proteins using mass spectrometry

The supernatant culture media (in serum free media) of ISC, ISC/R and Control CF cells following 3 days post treatment were concentrated using centrifugal filters of 3000 NMWL (Nominal Molecular Weight Limit) (Amicon Ultra-15). The concentrated proteins were quantified by standard BCA assay and 1.0 µg protein from each group (*n* = 3) was used to identify the protein contents employing LC MS/MS following our previous protocols (Thankam et al. 2022b) (Rappsilber et al. 2007). Raw data were validated using UniProt *Sus scrofa (Pig)* database and the average peak area of top three peptides in each protein in ISC and ISC/R groups were normalized to the control and were expressed as fold change (FC) in log2 scale. However, the difference was used for normalization if the average area was zero. The log2 FC and the cardiac function of the protein were considered for identifying the potential candidates based on their expression status in ISC and ISC/R groups. The major proteins identified were Cofilin 1, Calcitonin receptor-stimulating peptide-2 (CRSP2), Heat shock protein-27 (HSP27), HSP90, and interleukin-8 (IL8) based on their relative abundance in the supernatant which were used for further analysis.

### Histology

The harvested LV tissues were grouped into control MI (LV-MI-C), infarcted myocardium (LV-MI), ischemic (I) myocardium at the anastomosis site following CABG (LV-CABG) and non-ischemic myocardium from similar anatomic location of normal pigs (LV-CABG-C). The tissues were formalin fixed, paraffin embedded, sectioned onto microscopic slides, deparaffinized and used for histology. The histology was performed using pentachrome staining for LV tissues following our previously reported protocol (Thankam et al. 2018b). Also, the calcium deposits in the myocardial tissues were determined by the standard von Kossa staining as reported elsewhere (Schroeder et al. 2021). Xylene based mounting media was used to mount the stained slides and were imaged using a fluorescent slide scanner system (Leica, Thunder) at 20 × magnification.

### Tissue immunofluorescence

Expression level of the CF-secreted protein mediators identified using mass spectrometry including Cofilin 1, CRSP2, HSP27, HSP90 and IL8 along with the cardiac biomarker Troponin I and ROS homeostasis regulator Nrf2 were determined by immunofluorescence following our previously reported protocols (Thankam et al. 2016a, b). The deparaffinized tissues were used and antibodies against Cofilin 1 (ab42824), CRSP2 (ab196624), HSP27 (ab239499), HSP90 (ab13492), IL8 (ab106350), Troponin I (Trop I) (ab10231), and Nrf2 (ab92946) at a dilution of 1:200 and corresponding fluorochrome secondary antibodies at 1:400 dilution were used for the staining. Nuclei were counterstained with 4′,6-diamidino-2-phenylindole (DAPI) (H-1200), imaged using a fluorescent slide scanner system (Leica, Thunder) at 20 × magnification, mean fluorescence intensity (MFI) was quantified using ImageJ software, MFI was normalized to the number of cell nuclei and the results were expressed as log2 FC based on the normal control group (Thankam et al. 2022e). A negative control without the primary antibody was maintained in a similar manner to detect to fix the exposure time minimizing the background.

### Expression of CF-secreted mediators in cultured CF

The CF cells were cultured under ischemia and reperfusion as mentioned above and the expression status of Cofilin 1, CRSP2, HSP27, HSP90, IL8, Troponin I, and Nrf2 were determined at transcript, protein, and single cell levels. The experimental groups were CF-C, CF-ISC and CF-ISC/R respectively for the control, ischemic and reperfusion groups.

#### qRT-PCR

Trizole method was used to isolate total cellular RNA from each group of CF, reverse transcribed to cDNA using cDNA synthesis kit (AzuraQuant), mRNA transcripts for the genes Cofilin 1, CRSP2, HSP27, HSP90, IL8, Troponin I, and Nrf2 were amplified using specific set of primers (Table 1) and quantified by real-time PCR (Applied Biosystems, Waltham, MA) employing SYBR Green chemistry following our established protocols (Thankam et al. 2018a). 18s rRNA was used as a housekeeping reference gene and the transcript level of the genes were normalized to that of 18s rRNA and the results were represented as log_2_ fold-change (FC) with respect to control.

**Table 1:** Primers and sequence used for qRT-PCR

Primers	Sequence
Cofilin (F)	GCCAATTACTCGGATCCCGGC
Cofilin R	CCCGTCAGAGACAGCCACAC
HSP27 (F)	CGTCTCCCTGGACGTCAACC
HSP27 R	ACACCGGGAAATGAAGCCGT
IL-8 (F)	GGACCCCAAGGAAAAGTGGG
IL-8 R	AATTCTTGGGAGCCACGGAGA
TROP I (F)	GAGCCGCACGCCAAGAAAAA
TROP I R	CGCAATCTGCAGCATCAGGG
CRSP2 (F)	GTCTCTGCCGCTTCTTCCACA
CRSP2 R	AAAGGCAATCTCACGGGTGC
NRF2 (F)	CCTGTGGATGAGGCTCTACGG
NRF2 R	GGAACGGTCATTCCCATCCCA
HSP90 (F)	TCCTGCTGTACGAAACCGCT
HSP90 R	TAGATCCTGTTGGCGTGCGT
TROP I (F)	GAGCCGCACGCCAAGAAAAA
TROP I (R)	CGCAATCTGCAGCATCAGGG
I8s rRNA (F)	ACGTTGGCGAGAGCGTGG
I8s rRNA (R)	AGGTGGAGGAGGCGAGAGAG

#### Immunofluorescence

The CF were cultured in 8 well chamber slides and ISC and ISC/R were induced as mentioned above. The cells were formalin fixed for 1 h and immunostaining for Cofilin 1, CRSP2, HSP27, HSP90, IL8, Troponin I, and Nrf2 and the quantification was performed as mentioned above. The primary antibodies at a dilution of 1:300 and secondary antibodies at 1:500 was used.

#### Western blot

The cells were treated as mentioned above in T75 flasks, lysed using M-per lysis buffer (Cat# 78501, Thermo Fischer) and the total proteins were quantified using BCA assay for each group. For Western Blot analysis, 100µg sample was mixed with an equal volume of 2 × Laemmli buffer, denatured at 95°C, ran SDS-PAGE, transferred to PVDF membrane at 20V for 25 min (TRANS BLOT, Bio-Rad), blocked with 5% nonfat dry milk in PBS-T (Bio-Rad, Cat #1706404) for 3 h, incubated with primary antibodies CRSP2, HSP27, HSP90, IL8, Troponin I, and Nrf2 (1:1000 in PBS-T) overnight at 4°C, and imaged following the incubation with fluorochrome-conjugated secondary antibodies for 2 h. The band area was calculated using ImageJ software using ‘*Gels’* analysis mode, the protein expression was normalized to GAPDH (ab9484), and Log2 fold change based on control was calculated. Western blotting for CRSP2 was not performed due to the unavailability of swine-specific antibodies for WB.

#### Single cell RNA sequencing (scRNA-seq)

scRNAseq was performed for deciphering the expression profile at single cell dimension and the cellular phenotypes based on the upregulation of Cofilin 1, CRSP2, HSP27, HSP90, IL8, Troponin I, and Nrf2 in CF under ISC and ISC/R compared to control. Single-cell library preparation on the CF was performed using the Chromium Single Cell 5′ v2 chemistry at commercially available 10 × Genomics Chromium System (Children's Hospital Los Angeles SC2 Core, CA) capturing ~ 10,000 cells per sample following our previous protocols (Thankam and Agrawal 2021) (Thankam et al. 2022b). The processed raw data was assessed using sus_scrofa_11 database (https://uswest.ensembl.org/Sus_scrofa/Info/Index) for gene expression which was further analyzed using Loupe Browser 5.0.1. using graph-based analysis mode. The respective cell clusters expressing the genes of interest were analyzed in LibraryId mode filtered based on the extent of each gene expression which were sorted based on the log2 fold change to screen upregulated and downregulated genes. The phenotyping criteria was based on the maximum FC (Cofilin 1: FC > 5, CRSP2: FC > 2, HSP27: FC > 5, IL8: FC > 2, HSP90: FC > 5, and Nrf2: FC > 4) to secure the predominant sub-phenotypes based on the differentially expressed genes and to minimize the noise. Loupe Browser 5.0.1. was used to plot the t-SNE and violin plots whereas GraphPad Prism 9 was used to plot the scatter plots from the expression profile. In order to assess the differential expression, the gene expression profile was sorted based on the descending order of the FC of individual genes in the experimental group that mapped predominant cell density. The highly altered genes (FC > 2 and FC < 2) were assessed for the possible pathways, based on human genome using KEGG database, in the program PATHVIEW (https://pathview.uncc.edu) following the instructions provided. The pathways were generated automatically, according to the FC, by the program and were downloaded as png files (Luo and Brouwer 2013).

### Statistical analysis

The protein expression in LC MS/MS was represented as average ± standard deviation of log2 FC and the statistical significance was determined by Multiple *‘t’* tests using GraphPad Prism software 8.2.1 (441). The log2 FC for immunostaining, WB and qRT-PCR was expressed as mean ± SD and the statistical significance was evaluated by one way ANOVA using Tukey’s multiple comparison test employing. The tissue specimen that displayed background were omitted from the analysis. The mean for each specimen/experimental replicate was the average MFI of at least 3 images randomly acquired images which was used for calculating log2 FC in immunostaining. *P* < 0.05 was set as statistically significant for all experiments.

## Results

### Screening of supernatant proteins using mass spectrometry

**Fig. 1:**
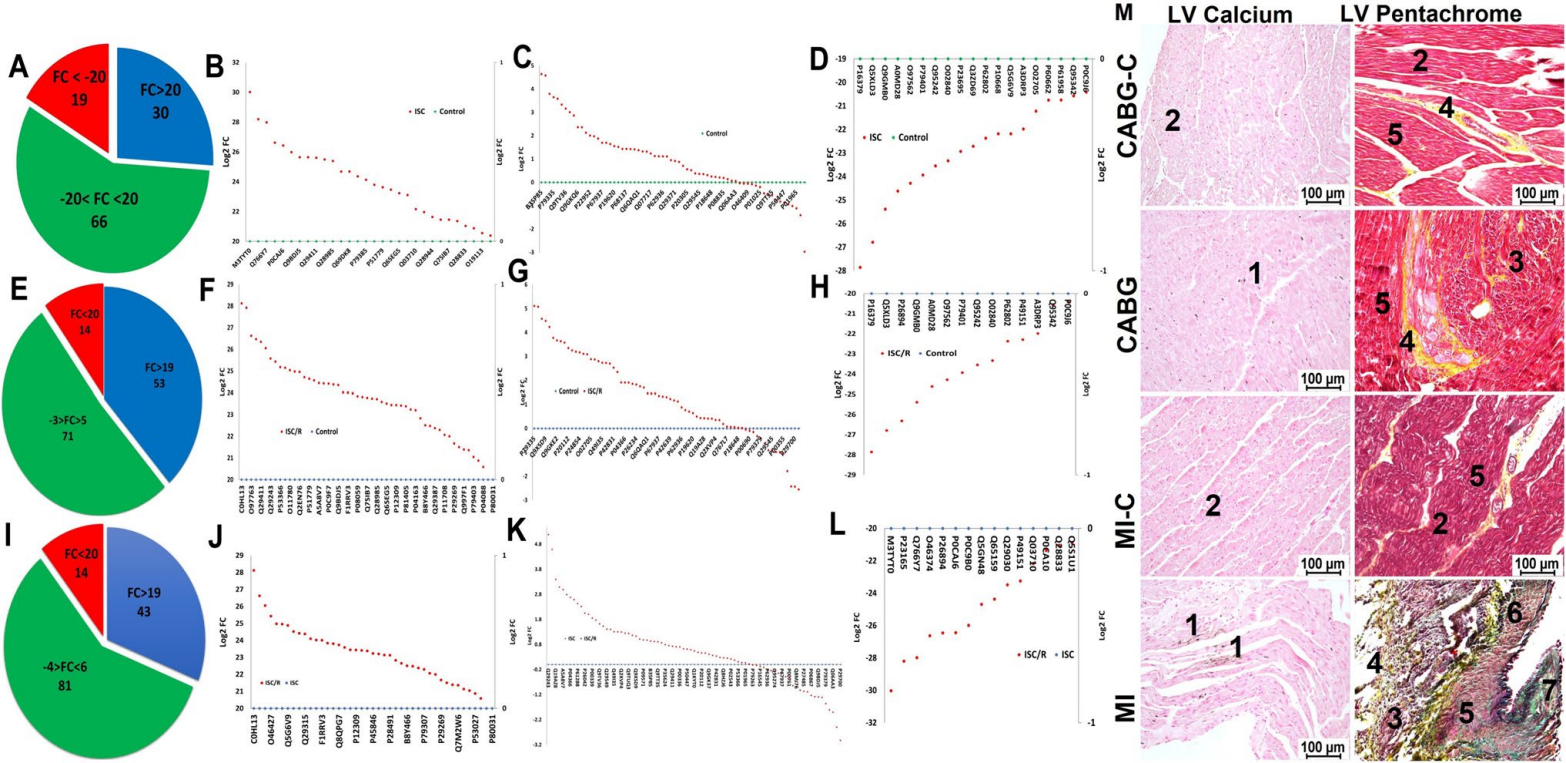
The level of secreted proteins of ischemia challenged CF using mass spectrometry. **A** Pie diagram showing the proportion of proteins based on the FC expression in the ISC vs Control groups, **B** Scatter diagram of newly upregulated proteins in ISC group which were absent in the control, **C** Scatter diagram showing the FC distribution of proteins expressed in both ISC and control groups. **D** Scatter diagram of newly downregulated proteins in ISC group which were present exclusively in the control. **E** Pie diagram showing the proportion of proteins based on the FC expression in the ISC/R vs Control groups, **F** Scatter diagram of newly upregulated proteins in ISC/R group which were absent in the control, **G** Scatter diagram showing the FC distribution of proteins expressed in both ISC/R and control groups. **H** Scatter diagram of newly downregulated proteins in ISC/R group which were present exclusively in the control. **I** Pie diagram showing the proportion of proteins based on the FC expression in the ISC vs ISC/R groups, **J** Scatter diagram of newly upregulated proteins in ISC group which were absent in the ISC/R, **K** Scatter diagram showing the FC distribution of proteins expressed in both ISC and ISC/R groups. **L** Scatter diagram of newly downregulated proteins in ISC group which were present exclusively in the ISC/R. **M** The histomorphology evaluations using von Kossa staining and Movat’s Pentachrome staining showing (1) calcification, (2) intact ECM, (3) ECM disorganization, (4) collagen deposition, (5) muscle, (6) elastic fibers, and (7) mucin

A total of 115 proteins were identified in the ISC group of CF cells in comparison to control with an overall log2 FC ranging from -28 to + 30 (Fig. 1A) (Supplementary Table 1). Among these, the FC of 30 proteins (26.09%) ranged from + 20 to + 30 (Fig. 1A and B), 66 proteins (57.39%) were in the range of –3 to + 5 (Fig. 1A and C), and 19 proteins (16.52%) were in the range of –20 to –27 (Fig. 1A and D). Interestingly, all 30 proteins that there upregulated with FC >  + 20 were absent expression in the control group (Fig. 1B). Similarly, 19 proteins that were downregulated with FC < –20 were completely absent in the ischemic group (Fig. 1D). Additionally, 138 proteins were identified in the ISC/R group in comparison to control with an overall log2 FC ranging from –28 to + 28 (Fig. 1E) (Supplementary Table 1). Among these, 53 proteins (38.41%) ranged from + 19 to + 28 (Fig. 1E and F), 71 proteins (51.45%) were in the range of –3 to + 5 (Fig. 1E and G), and 14 proteins (10.14%) ranged from –20 to –28 (Fig. 1E and H). Interestingly, the 53 proteins that were upregulated with FC >  + 19 were completely absent in the control group (Fig. 1E). Also, the 14 proteins that were downregulated with FC < –20 were completely absent in the ISC/R group (Fig. 1E). Moreover, 139 proteins were identified in the ISC/R group in comparison to the ISC group with an overall log2 FC ranging from –30 to + 28 (Fig. 1I) (Supplementary Table 1). Among these, 43 proteins (30.94%) ranged from + 19 to + 28 (Fig. 1I and J), 81 proteins (58.27%) ranged from –3 to + 5 (Fig. 1I and K), and 15 (10.79%) ranged from –20 to –30 (Fig. 1I and L). Interestingly, the 43 proteins that were upregulated with FC >  + 19 were completely absent in the ISC group (Fig. 1I). The major protein mediators identified based on their cardiac function is shown in the Table 2. Among the upregulated proteins CRSP2, HSP27, and IL-8 and among the downregulated proteins Cofilin-1, and HSP90 in the ISC group were considered for further studies. In addition, the cardiac biomarker Troponin-I and antioxidant regulator Nrf2 were included for detailed evaluations owing to their established pathological significance.

**Table 2:** Details of the major secreted protein mediators identified in the ISC group compared to the control (C) and ISC/R groups

Sl/No	Proteins	Log2 FC (Av.)	Cardiac functions	References
1	Rho associated protein kinase 2	C vs ISC (+ 30.02) C vs ISC/R (0.00) ISC vs ISC/R (-30.02)	Associated with Rho proteins Formation of atheroma	(Mallat et al. 2003)
2	Protein MGF 360-3L	C vs ISC (+ 28.2) C vs ISC/R (0.00) ISC vs ISC/R (-28.2)	Viral immunology	(Ramírez-Medina et al. 2020)
3	Calcitonin receptor stimulating peptide 2 (CRSP2)	C vs ISC (+ 27.98) C vs ISC/R (0.00) ISC vs ISC/R (-27.98)	Vasodialation in heart Calcium homeostasis	(Anand et al. 1991)
4	DNA Topoisomerase 2 alpha	C vs ISC (+ 26.63) C vs ISC/R (0.00) ISC vs ISC/R (-26.63)	DNA replication Cell cycle	(Järvinen et al. 1996)
5	Interleukin-8	C vs ISC (+ 27.98) C vs ISC/R (0.00) ISC vs ISC/R (-27.98)	Neutrophil chemotaxis Elevetes in unstable angina Earlier marker than CK-MB	(Kanda et al. 1996)
6	Uncharacterized protein C717R	C vs ISC (26.44) C vs ISC/R (ND) ISC vs ISC/R (-26.44)	Unknown	
7	Putative ATP-dependent RNA helicase	C vs ISC (+ 25.99) C vs ISC/R (0.00) ISC vs ISC/R (-25.99)	Unknown	
8	Dystrophin	C vs ISC (+ 24.61) C vs ISC/R (0.00) ISC vs ISC/R (-24.69)	Cytoskeletal component Decreased following MI	(Yoshida et al. 2003)
9	Putative poly(A) polymerase catalytic subunit	C vs ISC (+ 24.36) C vs ISC/R (0.00) ISC vs ISC/R (-24.36)	Transcription of poly adenylate mRNA Apotosis in cardiac reperfusion injury	(Li et al. 2014)
10	Kit ligand	C vs ISC (+ 23.47) C vs ISC/R (0.00) ISC vs ISC/R (-23.47)	Stem cell activation Cardiac healing Triggers growthfactor release	(Higuchi et al. 2009)
11	Vascular endothelial growth factor A	C vs ISC (+ 0.95) C vs ISC/R (-22.9) ISC vs ISC/R (-23.25)	Angiogenic growth factor Proliferation, migration, and permeability of endothelial cells Cardiac healing	(Kranz et al. 2000)
12	Complement factor B	C vs ISC (+ 22.17) C vs ISC/R (0.00) ISC vs ISC/R (-22.17)	Complement factor Pro-inflammatory Aggravates MI	(Singh et al. 2009)
13	Von Willebrand factor	C vs ISC (+ 20.88) C vs ISC/R (0.00) ISC vs ISC/R (-20.88)	Vascular hemostasis Upregulates in MI	(Jansson et al. 1991)
14	Heat shock protein 27	C vs ISC (+ 20.56) C vs ISC/R (0.00) ISC vs ISC/R (-20.88)	Intracellular stress protein Antioxidant response Insulin sensitivity Cardiac healing Cardiomyocyte integrity	(Xu et al. 2011)
15	Insulin-like growth factor II	C vs ISC (-22.93) C vs ISC/R (-1.79) ISC vs ISC/R (+ 21.14)	Growth hormone effect Improves cardiac healing Prevents apoptosis in CM	(Demetz et al. 2017)
16	Prelamin-A/C	C vs ISC (-22.71) C vs ISC/R (+ 0.43) ISC vs ISC/R (+ 23.14)	Disrupts mitosis Induces DNA damage Upregulates in ischemia	(Brayson et al. 2019)
17	Heat shock protein 90-alpha	C vs ISC (-21.22) C vs ISC/R (+ 2.89) ISC vs ISC/R (+ 21.10)	Molecular chaperone Protects from ischemic injury	(Aceros et al. 2019, p. 90)
18	Cofilin-1	C vs ISC (-22.18) C vs ISC/R (+ 2.80) ISC vs ISC/R (+ 24.98)	Identified as a potential Biomarker for acute MI	(Xu et al. 2019)

### LV tissues following MI and CABG sustains ischemic pathology

The von Kossa staining of LV tissues displayed increased calcium deposits in the LV-MI and moderate calcification in the LV-CABG tissues compared to their respective controls (Fig. 1M). The pentachrome staining revealed extreme ECM disorganization, fibrosis, limited muscle fibers, increased mucin deposition, and inflammation in the LV-MI tissues suggesting the severe ischemic injury. Interestingly, the LV-CABG tissues displayed ECM disorganization and collagen deposition along with muscle fibers suggesting inflammatory episodes; however, without mucin deposits (Fig. 1M). On the other hand, the control LV tissues displayed intact ECM without the histological features of inflammation and fibrosis (Fig. 1M).

### Response of the secreted mediators in the tissues and cells from ischemic myocardium

#### Ischemia decreased the expression of Cofilin 1 in the ischemic LV tissues and CF

**Fig. 2:**
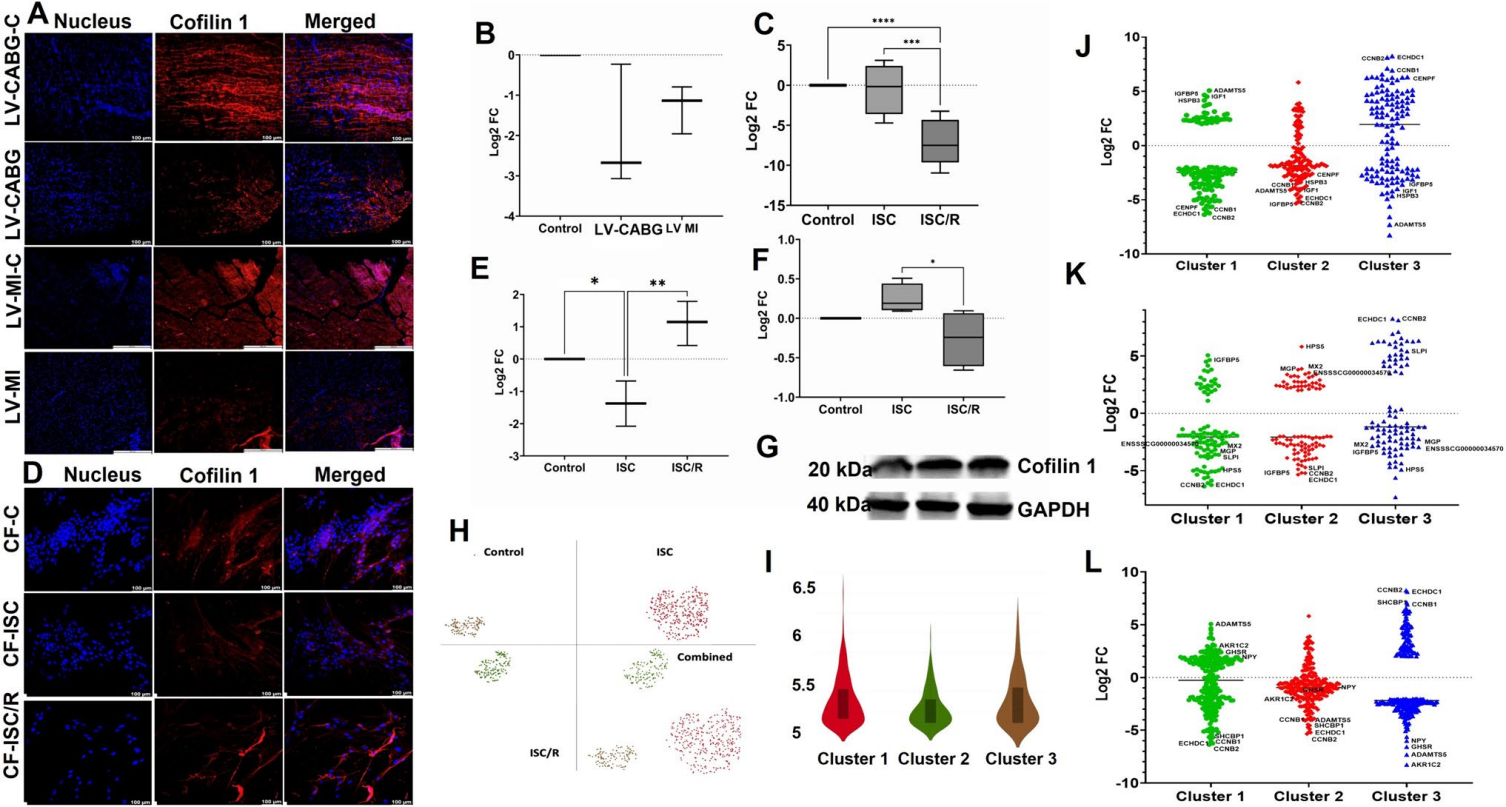
Representative images for the immunofluorescence analysis for the expression of Cofilin 1 in the LV (**A**) (*N* = 3) and the quantification of protein expression of Cofilin 1 in LV tissues calculated from MFI (**B**). **C** Quantification of Cofilin 1 gene expression in CF (*N* = 8) following the treatment with ISC and ISC/R using qRT-PCR relative to the housekeeping gene GAPDH normalized to the control and presented as Log2 FC. Representative images for the immunofluorescence and quantification for the expression of Cofilin 1 in the CF (*N* = 3) following the treatment with ISC and ISC/R (**D** and **E**). Representative image of Western blot and quantification showing the expression status of Cofilin 1 in the ISC and ISC/R groups compared to the control in CF (*N* = 4) (**F** and **G**). Single cell genomics of Cofilin 1 + CF showing the (**H**) (split and combined views of Control, ISC and ISC/R groups of t-SNE plot) showing the distribution of cells within all clusters based on the global expression of 4601 genes. Violin plot (**I**) (FC 5 and 5.5) indicating the alteration of genes in the 3 clusters. **J** Scatter plot FC expression indicating the alteration of Cluster 1 genes reflecting the contrasting phenotype compared to Clusters 2 and 3. **K** Scatter plot FC expression indicating the alteration of Cluster 2 genes reflecting the contrasting phenotype compared to Clusters 1 and 3. **L** Scatter plot FC expression indicating the alteration of Cluster 3 genes reflecting the contrasting phenotype compared to Clusters 1 and 2. (**** P < 0.0001, *** P < 0.001, ** P < 0.01, * P < *0.05 and unlabeled groups represent NS*)

The level of Cofilin-1 was decreased in both LV-CABG (*P* = 0.0943) and LV-MI (*P* = 0.2913) groups compared to the control; however, was statistically not significant. Also, the variation between LV-CABG and LV-MI groups were statistically not significant (Fig. 2A and B). The mRNA transcripts of Cofilin-1 were significantly decreased in the ISC/R CF (*P* < 0.0001) compared to the control whereas the decrease in the ISC group (*P* = 0.8768) was statistically not significant. The extent of decrease was significantly more in the ISC/R group compared to the ISC group (*P* = 0.0002) (Fig. 2C). Similarly, the level of Cofilin-1 was significantly decreased in the ISC group (*P* = 0.0552) of cultured CF and decreased non-significantly in the ISC/R group (*P* = 0.1127) compared to the control. Cofilin-1 was significantly decreased in the ISC group than ISC/R group (*P* = 0.0040) as evident from immunostaining (Fig. 2D and E). Western Blot analysis displayed upregulation of Cofilin-1 in ISC (*P* = 0.3360) CF with concomitant downregulation in ISC/R (*P* = 0.2935) CF compared to the control; however, were statistically not significant. Notably, ISC/R displayed significant downregulation of Cofilin-1 compared to the ISC (*P* = 0.0308) CF (Fig. 2F and G).

##### Cofilin-1 + Cluster 1 CF represent a pro-survival phenotype

Single cell genomics revealed that 622 CF cells were positive for Cofilin-1 (FC > 5), which constituted 2.07% of the parent population where 120 cells were in the control group (19.13% of Cofilin-1 + population), 352 cells in the ISC group (56.59% of Cofilin-1 + population), and 150 cells in the ISC/R group (23.95% of Cofilin-1 + population). Overall, the Cofilin-1 + cells favored the ISC group and were mapped in 3 cluster based on the expression level of 4601 genes (Fig. 2H and I). Cluster-1 displayed 352 cells where 100% cells were displayed in the ISC CF, and the violin plot revealed the distribution of 4601 genes in cluster 1 CF based on expression status in ISC group (Fig. 2H and I) (Supplementary File 1). ADAMTS5 (ADAM metallopeptidase with thrombospondin type 1 motif 5) (FC + 5.07, *P* < 0.0001), IGFBP5 (insulin like growth factor binding protein 5) (FC + 4.66, *P* < 0.0001) and IGF1 (insulin like growth factor 1) (FC + 4.51, *P* < 0.0001) were the major genes highly altered in the ISC group of Cluster 1 (Fig. 2J) (Supplementary File 1). On the other hand, CCNB1 (Cyclin B1) (FC –6.08, *P* < 0.0001), ECHDC1 (ethyl-malonyl-CoA decarboxylase 1) (FC –6.24, *P* < 0.0001), and CCNB2 (Cyclin B2) (FC –6.37, *P* < 0.0001) were the major genes highly altered in the cluster 1 compared to the other clusters (Fig. 2J) (Supplementary File 1). Interestingly, most of the upregulated genes in the cluster 1 cells were significantly downregulated in the Clusters 2 and 3 (and vice versa) reflecting the contrasting phenotypes (Fig. 2J). The pathway analysis of Cofilin-1 + Cluster 1 cells displayed multiple pathways associated with (1) calcium homeostasis with a concomitant operation of cell proliferation, metabolism, and exocytosis (Supplementary Fig. 1A), (2) ischemia tolerance via HIF signaling accelerating the matrix function, oxygen delivery, angiogenesis, and anabolic pathways with a concomitant reduction in oxygen consuming pathways (Supplementary Fig. 1B), and (3) p53 mediated cell survival (Supplementary Fig. 1C). Overall, Cofilin-1 + Cluster 1 cells represents a pro-survival/regenerative phenotype.

##### Cofilin-1 + Cluster 2 CF signify a pro-inflammatory phenotype

Cluster 2 CF displayed 150 cells where 99.33% cells were mapped in the ISC/R group (Fig. 2H and I) (Supplementary File 1). HPS5 (Hermansky-Pudlak syndrome-5) (FC + 5.82, *P* < 0.0001), MGP (matrix gla protein) (FC + 3.89, *P* < 0.0001), and MX2 (MX dynamin like GTPase 2) (FC + 3.81, *P* < 0.0001) were significantly upregulated in the ISC/R group of Cofilin-1 + Cluster 2 CF compared to the Clusters 1 and 3 (Fig. 2K) (Supplementary File 1). The major downregulated genes were CCNB2 (FC –4.66, *P* < 0.0001), ECHDC1 (FC –6.24, *P* < 0.0001), and IGFBP5 (insulin like growth factor binding protein 5) (FC –6.38, *P* < 0.0001) in the cluster 2 compared to the other two clusters (Fig. 2K) (Supplementary File 1). Interestingly, most of the upregulated genes in the cluster 2 cells were significantly downregulated and vice versa in the Clusters 1 and 3 suggesting the contrasting phenotypes (Fig. 2K). The pathway analysis of Cofilin-1 + Cluster 2 cells displayed the inflammatory signaling associated with (1) chemokine signaling such as chemotaxis, cell migration, and ROS production (Supplementary Fig. 1D), (2) TNF signaling accelerating the apoptosis and leukocyte activation (Supplementary Fig. 1E), and (3) NOD-like signaling pathways aggravating the pro-inflammatory responses (Supplementary Fig. 1F). Overall, Cofilin-1 + Cluster 2 cells represents a pro-inflammatory phenotype.

##### Cofilin-1 + Cluster 3 CF reflect a proliferative phenotype

More than 99% of the Cluster 3 cells were mapped in the control group (Fig. 2H and I) (Supplementary File 1). CCNB2 (FC + 8.26, *P* < 0.0001), ECHDC1 (FC + 8.13, *P* < 0.0001), and SHCBP1 (SHC binding, and spindle associated 1) (FC + 7.12, *P* < 0.0001) were significantly upregulated in the ISC/R group of Cofilin-1 + Cluster 3 CF compared to Clusters 2 and 3 (Fig. 2L) (Supplementary File 1). The major downregulated genes were GHSR (growth hormone secretagogue receptor) (FC –6.57, *P* < 0.0001), ADAMTS5 (FC –7.23, *P* < 0.0001), and AKR1C2 (aldo–keto reductase family 1 member C2) (FC –8.26, *P* < 0.0001) in the cluster 3 compared to the other two clusters (Fig. 2L) (Supplementary File 1). Interestingly, most of the upregulated genes in the cluster 3 cells were significantly downregulated and vice versa in the Clusters 1 and 3 suggesting the contrasting phenotypes (Fig. 2L). The pathway analysis of Cofilin-1 + Cluster 3 cells displayed the survival responses including (1) PI3K-AKT signaling mediated through the increased protein synthesis, glucose metabolism, cell proliferation and repair (Supplementary Fig. 1G), and (2) cell cycle progression (Supplementary Fig. 1H). Overall, Cofilin-1 + Cluster 3 cells represents a proliferative phenotype whereas the Clusters 2 and 3 represent non-proliferative and secretory phenotypes.

### Ischemia increased the expression of CRSP2 in the ischemic LV tissues and CF

**Fig. 3:**
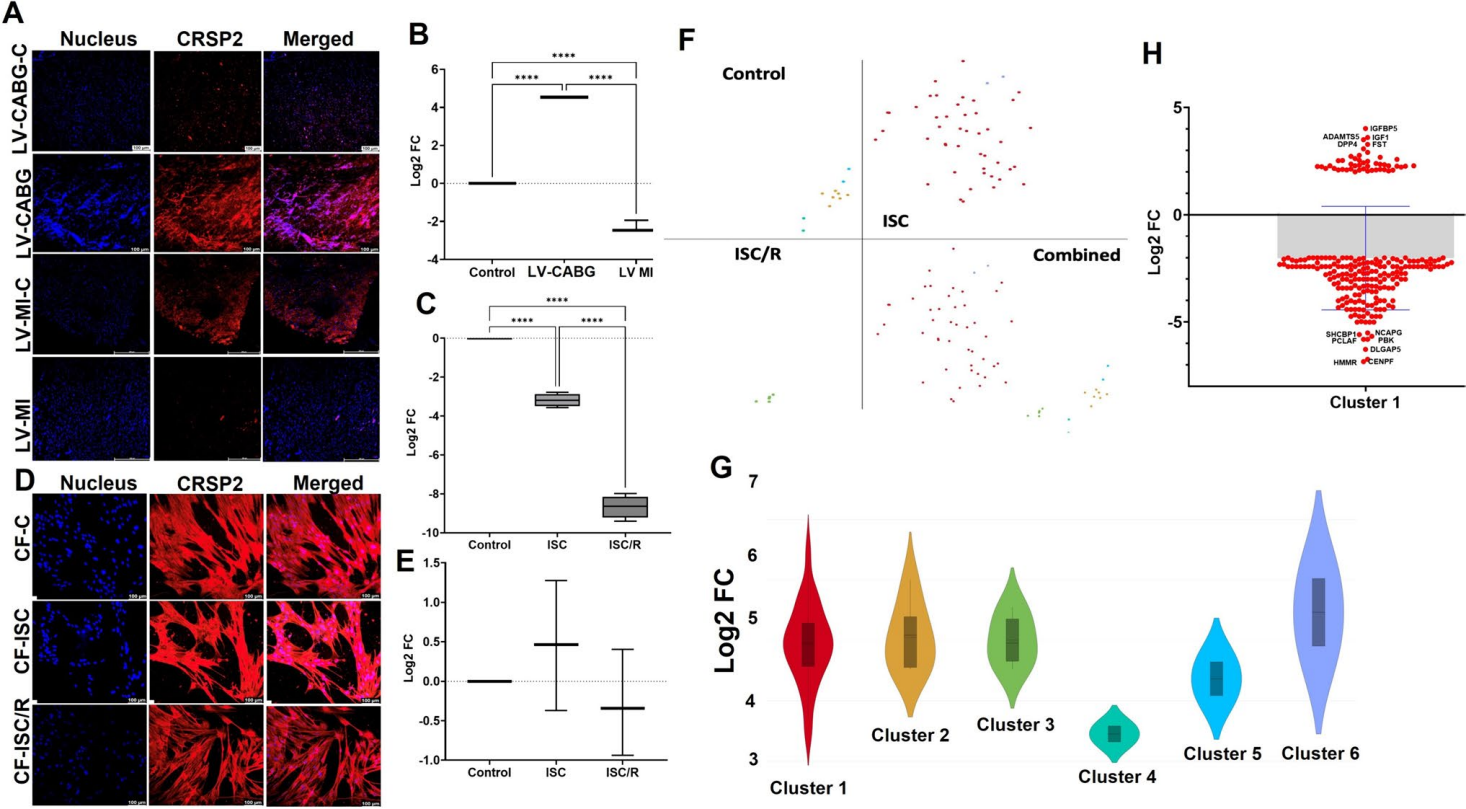
Representative images for the immunofluorescence analysis for the expression of CRSP2 in the LV (**A**) (*N* = 3) and the quantification of protein expression of CRSP2 in LV tissues calculated from MFI (**B**). **C** Quantification of CRSP2 gene expression in CF (*N* = 4) following the treatment with ISC and ISC/R using qRT-PCR relative to the housekeeping gene GAPDH normalized to the control. Representative images for the immunofluorescence and quantification for the expression of CRSP2 in the CF (*N* = 3) following the treatment with ISC and ISC/R (**D** and **E**). Single cell genomics of CRSP2 + CF showing the (**F**) (split and combined views of Control, ISC and ISC/R groups of t-SNE plot) showing the distribution of cells within all clusters based on the global expression of 6821 genes. Violin plot (**G**) (FC > 2) indicating the alteration of genes in the 6 clusters. **J** Scatter plot FC expression indicating the alteration of Cluster 1 genes reflecting the expression of the key signature genes. (**** P < *0.0001, and unlabeled groups represent NS*)

CRSP2 was significantly increased in LV-CABG (*P* < 0.0001) and LV MI tissues (*P* < 0.0001) compared to control. Also, the variation between LV-CABG and LV-MI groups were statistically significant (*P* < 0.0001) (Fig. 3A and B). The mRNA expression of CRSP2 was significantly decreased in both ISC (*P* < 0.0001) and ISC/R (*P* < 0.0001) CF compared to the control and ISC/R CF exhibited a significant reduction in CRSP2 transcripts than ISC CF (*P* < 0.0001) (Fig. 3C). Similar trend was observed in the CF cells in ISC and ISC/R group as evident from immunostaining (Fig. 3D and E). The level of CRSP2 protein was increased in the ISC CF (*P* = 0.6524) and decreased in the ISC/R group (*P* = 0.8335) compared to the control; however, was statistically not significant. Also, the alteration between ISC and ISC/R groups (*P* = 0.3566) were statistically not significant (Fig. 3D and E).

#### CRSP2 + Cluster 1 CF suggest a non-proliferative phenotype

Single cell genomics revealed that 63 CF were positive for CRSP2 (FC > 2), which constituted 0.2094% of the parent population where 45 cells in the ISC group (71.43% of CRSP2 + population) and the CRSP2 + cells favored the ISC group and were mapped in six clusters based on the expression status of 6821 genes (Fig. 3F and G). IGFBP5 (FC + 4.02, *P* < 0.0001), IGF1 (FC + 3.60, *P* < 0.0001), and ADAMTS5 (FC = 3.50, *P* < 0.0001) were the most significantly upregulated genes in the ISC group of CF (Fig. 3H) (Supplementary File 1). CENPF (centromere protein F) (FC –6.85, *P* < 0.0001), HMMR (hyaluronan mediated motility receptor) (FC –6.74, *P* = 0.0001), and DLGAP5 (DLG associated protein 5) (FC –6.28, *P* = 0.0001) were the most significantly downregulated genes (Fig. 3H) (Supplementary File 1). The pathway analysis of CRSP2 + Cluster 1 cells displayed the downregulation multiple key mediators of cell cycle pathways suggesting a non-proliferative phenotype (Supplementary Fig. 2A).

### The level of HSP27 was higher in the ischemic LV tissues and CF

**Fig. 4:**
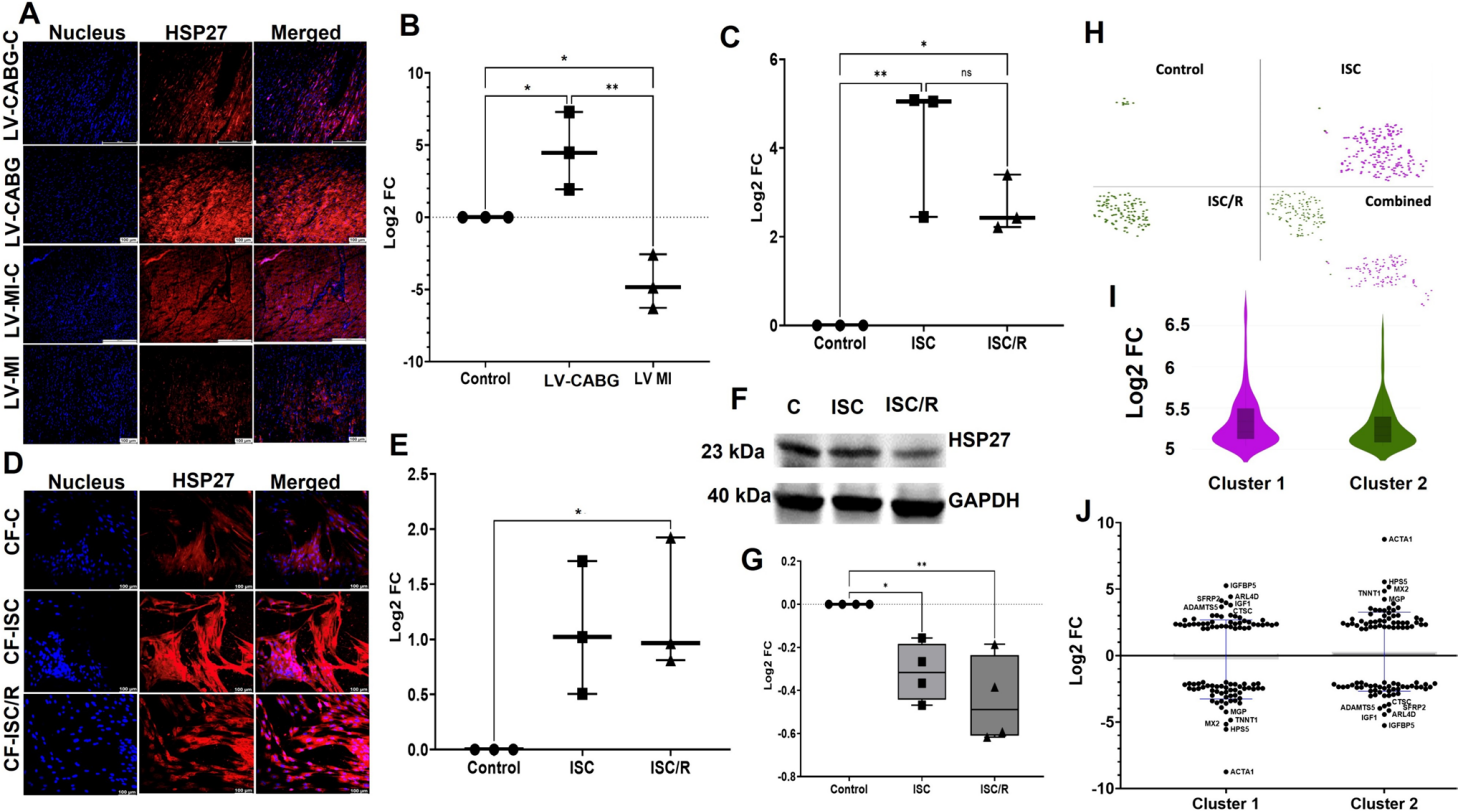
Representative images for the immunofluorescence analysis for the expression of HSP27 in the LV (**A**) (*N* = 3) and the quantification of protein expression of HSP27 in LV tissues calculated from MFI (**B**). **C** Quantification of HSP27 gene expression in CF (*N* = 3) following the treatment with ISC and ISC/R using qRT-PCR relative to the housekeeping gene GAPDH normalized to the control. Representative images for the immunofluorescence and quantification for the expression of HSP27 in the CF (*N* = 3) following the treatment with ISC and ISC/R (**D** and **E**). Representative image of Western blot and quantification showing the expression status of HSP27 in the ISC and ISC/R groups compared to the control in CF (*N* = 4) (**F** and **G**). Single cell genomics of HSP27 + CF showing the (**H**) (split and combined views of Control, ISC and ISC/R groups of t-SNE plot) showing the distribution of cells within all clusters based on the global expression of 6821 genes. Violin plot (**I**) (FC > 5) indicating the alteration of genes in the 2 clusters. **J** Scatter plot FC expression indicating the alteration of Cluster 1 and 2 genes reflecting the contrasting level of expression of the key signature genes. (* P < 0.05, ** P < *0.01, and unlabeled groups represent NS*)

HSP27 was significantly increased in LV-CABG (*P* = 0.0570) and significantly decreased in LV-MI (*P* = 0.0566) groups compared to the respective controls and LV-CABG displayed a significant increase in HSP27 compared to LV-MI (*P* = 0.0025) (Fig. 4A and B). The transcript levels of HSP27 in CF were significantly increased in ISC (*P* = 0.0039) and ISC/R (*P* = 0.0307) group compared to the control whereas the increase in ISC CF was statistically not significant compared to the ISC/R (*P* = 0.2040) CF (Fig. 4C). Immunostaining revealed the increased level of HSP27 in both ISC (*P* = 0.0809) and ISC/R (*P* = 0.0501) CF compared to the control; however, was statistically not significant in the ISC group. Also, the alteration between ISC and ISC/R groups (*P* = 0.9230) was statistically not significant (Fig. 4D and E). Western blot analysis revealed that HSP27 was significantly downregulated in both ISC (*P* = 0.0271), and ISC/R (*P* = 0.0038) CF compared to controls and the alteration between ISC, and ISC/R groups (*P* = 0.9853) was statistically not significant (Fig. 4F and G).

#### HSP27 + CF represent contrasting phenotypes

Single cell genomics revealed that 334 CF were positive for HSP27 (FC > 5), which constituted 1.1100% of the parent population where 10 cells were in the control group (2.99% of HSP27 + population), 196 cells in the ISC group (58.68% of HSP27 + population), and 124 cells in the ISC/R group (37.13% of HSP27 + population). Overall, the HSP27 + CF cells favored the ISC group and were mapped in two clusters (Fig. 4H and I). Cluster 1 CF displayed 196 cells where 100% cells were mapped in the ISC group, and the violin plot revealed the distribution of 4144 genes in HSP27 + CF based on expression status in ISC group (Fig. 4H and I) (Supplementary File 1). IGFBP5 (FC + 5.26, < 0.0001), ARL4D (ADP ribosylation factor like GTPase 4D) (FC + 4.43, *P* < 0.0001), and IGF1 (FC + 4.14, *P* < 0.0001) were the key genes significantly upregulated in the ISC group (Fig. 4J) (Supplementary File 1). ACTA1 (Actin, alpha skeletal muscle) (FC –8.75, *P* < 0.0001), HPS5 (FC –5.55, *P* < 0.0001), and MX2 (FC –5.16, *P* < 0.0001) were the most significantly downregulated genes (Fig. 4J) (Supplementary File 1). The pathway analysis of HSP27 + Cluster 1 cells displayed the (1) chemokine-receptor signaling (Supplementary Fig. 2B), (2) chemokine activation for cell migration, apoptosis, and ROS production (Supplementary Fig. 2C), and (3) PI3K-AKT signaling for cell survival suggesting a pro-survival phenotype (Supplementary Fig. 2D). Surprisingly, the HSP27 + Cluster 2 cells displayed exactly contrasting Log2 FC values for the same set of genes displayed by HSP27 + Cluster 1 cells and favored ISC/R group suggesting an anti-survival phenotype (Fig. 4J) (Supplementary File 1).

### Ischemic insults resulted in the downregulation of IL8 in the LV tissues and CF

**Fig. 5:**
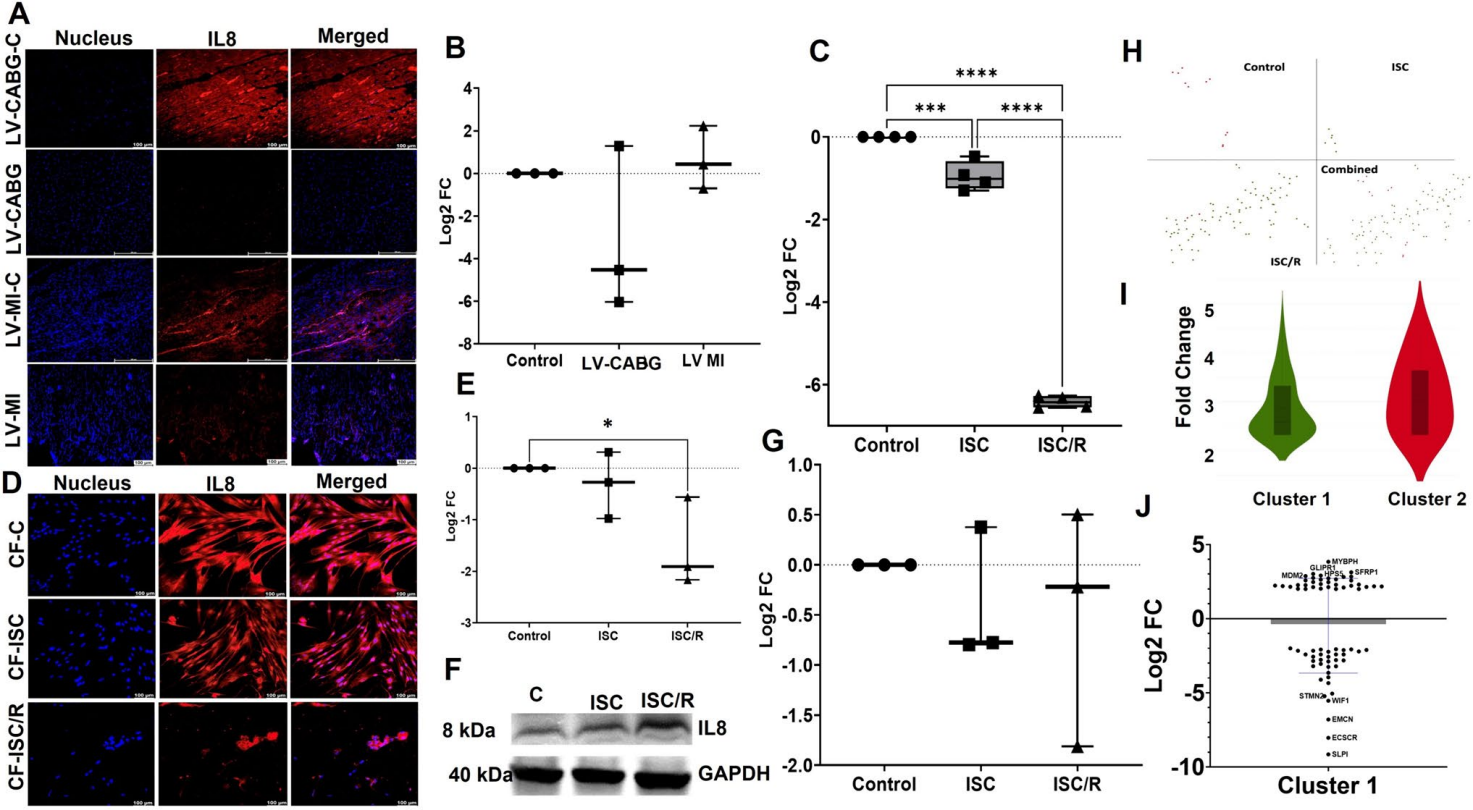
Representative images for the immunofluorescence analysis for the expression of IL8 in the LV (**A**) (*N* = 3) and the quantification of protein expression of IL8 in LV tissues calculated from MFI (**B**). **C** Quantification of IL8 gene expression in CF (*N* = 4) following the treatment with ISC and ISC/R using qRT-PCR relative to the housekeeping gene GAPDH normalized to the control. Representative images for the immunofluorescence and quantification for the expression of HSP27 in the CF (*N* = 3) following the treatment with ISC and ISC/R (**D** and **E**). Representative image of Western blot and quantification showing the expression status of IL8 in the ISC and ISC/R groups compared to the control in CF (*N* = 3) (**F** and **G**). Single cell genomics of IL8 + CF showing the (**H**) (split and combined views of Control, ISC and ISC/R groups of t-SNE plot) showing the distribution of cells within all clusters based on the local expression of 2654 genes. Violin plot (**I**) (FC > 2) indicating the alteration of genes in the 2 clusters. **J** Scatter plot FC expression indicating the alteration of Cluster 1 genes reflecting the expression of key signature genes. (* P < 0.05, *** P < 0.001, **** P < *0.0001, and unlabeled groups represent NS*)

The level of IL8 was decreased in LV-CABG (*P* = 0.3214) and increased in LV-MI (*P* = 0.9408) compared to control; however, the alterations were statistically not significant. Also, the variation between LV-CABG and LV-MI (*P* = 0.2134) groups were statistically not significant (Fig. 5A and B). The gene expression of IL8 was significantly decreased in both ISC (P = 0.0005), and ISC/R (*P* < 0.0001) CF compared to the control and ISC/R CF exhibited a significant reduction in IL8 transcripts than ISC CF (*P* < 0.0001) (Fig. 5C). The level of IL8 was decreased in both ISC (*P* = 0.8160), and ISC/R (*P* = 0.0514) CF compared to the control as evident from immunostaining where the level of IL8 was statistically significant in the ISC/R group. However, the alteration between ISC and ISC/R groups (*P* = 0.1119) was statistically not significant (Fig. 5D and E). Also, IL8 was downregulated in both ISC (*P* = 0.8138), and ISC/R (*P* = 0.7191) CF as determined by the Western blotting compared to the control; however, was not significant. Also, the variation between ISC and ISC/R groups (*P* = 0.9836) was statistically not significant (Fig. 5F and G).

#### IL8 + Cluster 1 CF reflect a proliferative pro-survival phenotype

Single cell genomics revealed that 99 CF cells were positive for IL8 (FC > 2), which constituted 0.3290% of the parent population where 11 cells were in the control group (12.22% of IL8 + population), 6 cells in the ISC group (6.67% of IL8 + population), and 80 cells in the ISC/R group (88.89% of IL8 + population). Overall, the IL8 + cells favored the ISC/R group and were mapped in two clusters (Fig. 5H and I). Cluster 1 displayed 86 CF cells where 6.97% cells were mapped in the ISC group, and 93.02% cells with the distribution of 2654 genes in the cluster 1 CF based on expression status in ISC/R group (Fig. 5H and I) (Supplementary File 1). MYBPH (Myosin Binding Protein H) (FC + 3.84, *P* = 0609), SFRP1 (Secreted frizzled-related protein 1) (FC + 3.31, *P* = 0609), and GLIPR1 (FC + 3.02, *P* = 0.0321) were the key genes significantly upregulated in the ISC/R group; however, the expression status of MYBPH and SFRP1 were statistically not significant (Fig. 5J) (Supplementary File 1). SLP1 (secretory leukocyte peptidase inhibitor) (FC –9.15, *P* = 0.0168), ECSCR (endothelial cell surface expressed chemotaxis and apoptosis regulator) (FC –8.03, *P* = 0.0026), and EMCN (endomucin) (FC –6.80, *P* = 0.0210) were the most significantly downregulated genes (Fig. 4J) (Supplementary File 1). The pathway analysis of IL8 + Cluster 1 cells displayed the (1) MAPK signaling activating cell proliferation and differentiation (Supplementary Fig. 2E), (2) cytokine-cytokine receptor interaction (Supplementary Fig. 2F), and (3) PI3K-AKT signaling for cell survival suggesting a proliferative pro-survival phenotype (Supplementary Fig. 2G).

### Ischemic insults resulted in the upregulation of HSP90 in the LV tissues and CF

**Fig. 6:**
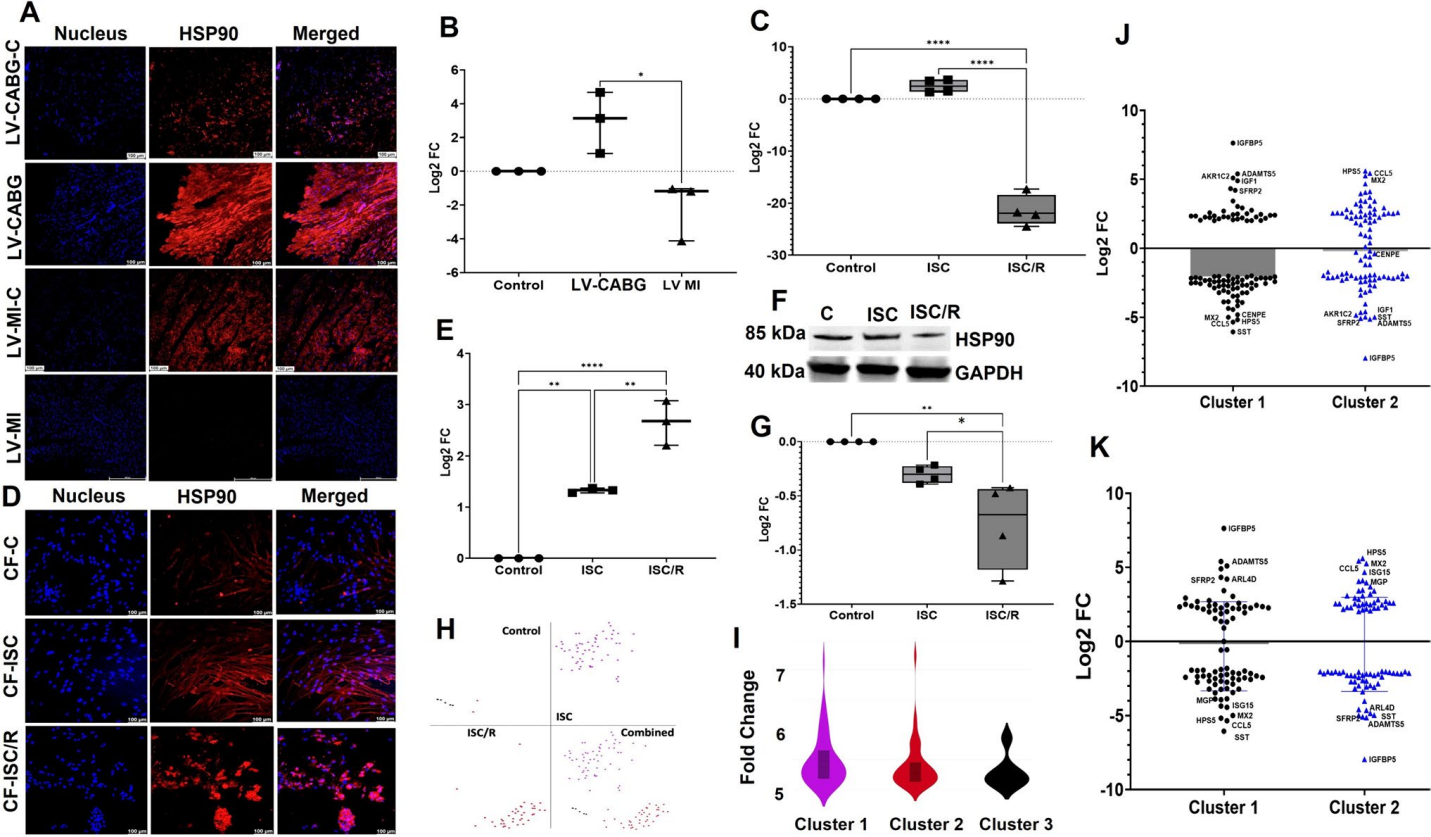
Representative images for the immunofluorescence analysis for the expression of HSP90 in the LV (*N* = 3) (**A**) and the quantification of protein expression of HSP90 in LV tissues calculated from MFI (**B**). **C** Quantification of HSP90 gene expression in CF (*N* = 4) following the treatment with ISC and ISC/R using qRT-PCR. Representative images for the immunofluorescence and quantification for the expression of HSP90 in the CF (*N* = 3) (**D** and **E**) (*N* = 3) following the treatment with ISC and ISC/R. **F** Representative image of Western blot and (**G**) quantification showing the expression status of HSP90 in the ISC and ISC/R groups compared to the control in CF (*N* = 4). Single cell genomics of HSP90 + CF showing the (**H**) (split and combined views of Control, ISC and ISC/R groups of t-SNE plot) showing the distribution of cells within all clusters based on the local expression of 4272 genes. Violin plot (**I**) (FC > 5) indicating the alteration of genes in the 2 clusters. **J** Scatter plot FC expression indicating the alteration of Cluster 1 genes reflecting the expression of key signature genes. (* P < 0.05, ** P < 0.01, **** P < *0.0001, and unlabeled groups represent NS*)

HSP90 was decreased in LV-CABG (*P* = 0.1032) and increased in LV-MI (*P* = 0.2516) compared to the control; however, was statistically not significant. Interestingly, LV-CABG displayed significantly higher level of HSP90 compared to LV-MI (*P* = 0.0123) (Fig. 6A and B). The transcript level of HSP90 (*P* = 0.2038) was increased in the ISC; however, was statistically not significant and was significantly decreased ISC/R (*P* < 0.0001) CF compared to the control. Also, the ISC/R displayed statistically significant decrease in HSP90 mRNA level compared to ISC (*P* < 0.0001) CF (Fig. 6C). Immunostaining revealed significant increase of HSP90 in both the ISC (*P* = 0.0016) and ISC/R (*P* < 0.0001) groups compared to the control CF, and the level of HSP90 was significantly decreased in the ISC/R (*P* < 0.0001) groups compared to the ISC CF (*P* = 0.0016) (Fig. 6D and E). Interestingly, HSP90 was downregulated in both ISC (*P* = 0.2196), and ISC/R (*P* = 0.0033) CF compared to the control; however, was not statistically significant in the ISC cells as evident from Western blotting. Also, the level of HSP90 was significantly decreased in the ISC/R groups compared to the ISC CF (*P* = 0.0511) (Fig. 6F and G).

#### HSP90 + Cluster 1 CF reflect pro-survival and Cluster 2 CF reflect pro-inflammatory phenotypes

Single cell genomics revealed that 102 cells were positive for HSP90 (FC > 5), which constituted 0.32% of the parent population where 55 cells were mapped in the ISC group (57.29% of HSP90 + population), and 41 cells in the ISC/R group (40.63% of HSP90 + population). Overall, the HSP90 + cells favored the ISC group and were mapped in 3 clusters based on the expression status of 4272 genes (Fig. 6H and I). Cluster 1 displayed 55 cells where 100% cells were mapped in the ISC group. IGFBP5 (FC + 7.63, < 0.0001), ADAMTS5 (FC + 5.40, *P* = 0.0009), AKR1C2 (FC + 5.10, *P* = 0.0060), and IGF1 (FC + 4.90, *P* < 0.0001) were the key genes significantly upregulated in the ISC group (Fig. 6J) (Supplementary File 1). SST (somatostatin) (FC –6.06, *P* = 1.0000), HPS5 (FC –5.35, *P* < 0.0001), CCL5 (FC –5.18, *P* = 0.9050), and MX2 (FC –5.00, *P* = 0.0001) were the most downregulated genes; however, was statistically not significant for SST and CCL5 (Fig. 6J) (Supplementary File 1). The pathway analysis of HSP90 + Cluster 1 cells displayed the downregulation of key inflammatory mediators in the pathways associated with (1) cytokine—cytokine-receptor signaling (Supplementary Fig. 3A), (2) NLR signaling (Supplementary Fig. 3B), and (3) TNF signaling suggesting a pro-survival phenotype (Supplementary Fig. 3C). Cluster 2 displayed 41 cells where 95.12% cells were mapped in the ISC/R group. HPS5 (FC + 5.61, < 0.0001), CCL5 (FC + 5.44, *P* = 0.7275), MX2 (FC + 5.26, *P* < 0.0001), and IGF1 (FC + 4.90, *P* < 0.0001) were the key genes upregulated in the ISC group; however, the upregulation in CCL5 was not significant (Fig. 6K) (Supplementary File 1). IGFBP5 (FC –7.96, *P* < 0.0001), ADAMTS5 (FC –5.14, *P* = 0.0049), SST (FC –5.09, *P* = 1.0000), and ARLD (FC –4.99, *P* < 0.0001) were the most downregulated genes; however, was statistically not significant for SST (Fig. 6K) (Supplementary File 1). The pathway analysis of HSP90 + Cluster 2 cells displayed the (1) cytokine—cytokine-receptor signaling (Supplementary Fig. 3A), (2) NLR signaling (Supplementary Fig. 3B), and (3) TNF signaling suggesting a pro-inflammatory phenotype (Supplementary Fig. 3C) as in Cluster 1.

### Ischemic insults resulted in the decreased level of Nrf2 in the LV tissues and CF

**Fig. 7:**
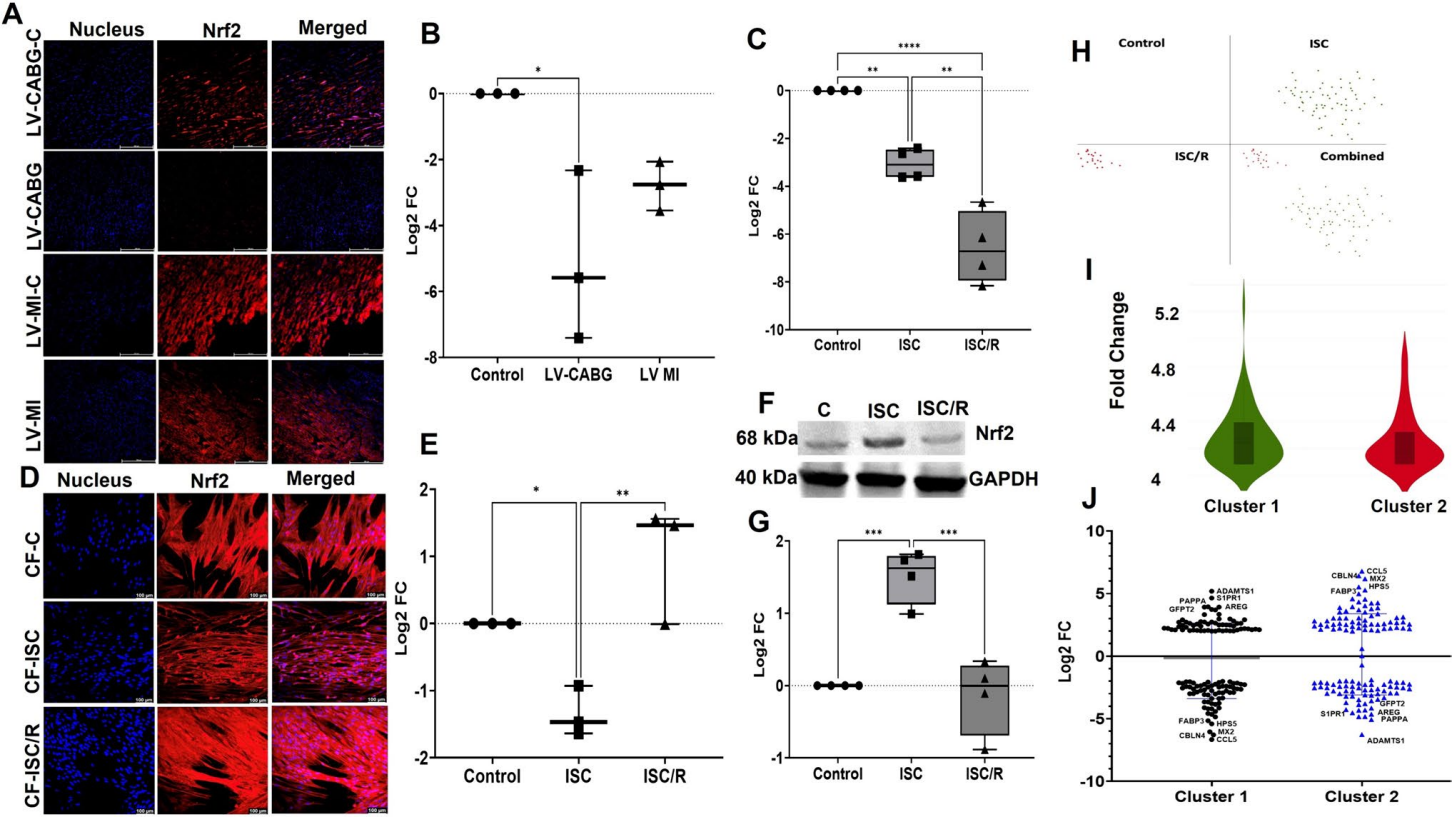
Representative images for the immunofluorescence analysis for the expression of Nrf2 in the LV (*N* = 3) (**A**) tissues and (**B**) the quantification of protein expression of Nrf2 in LV tissues calculated from MFI. **C** Quantification of Nrf2 gene expression in CF (*N* = 4) following the treatment with ISC and ISC/R using qRT-PCR. Representative images for the immunofluorescence and quantification for the expression of Nrf2 in the CF (*N* = 3) (**D** and **E**) following the treatment with ISC and ISC/R. **F** Representative image of Western blot and (**G**) quantification showing the expression status of Nrf2 in the ISC and ISC/R groups compared to the control in CF (*N* = 4). Single cell genomics of Nrf2 + CF showing the (**H**) (split and combined views of Control, ISC and ISC/R groups of t-SNE plot) showing the distribution of cells within all clusters based on the local expression of 5817 genes. Violin plot (**I**) (FC > 4) indicating the alteration of genes in the 2 clusters. **J** Scatter plot FC expression indicating the alteration of Cluster 1 genes reflecting the expression of key signature genes. (* P < 0.05, ** P < 0.01, *** P < 0.001, **** P < *0.0001, and unlabeled groups represent NS*)

Nrf2 was decreased in LV-CABG (*P* = 0.0159) and LV-MI (*P* = 0.1477) compared to the control; however, the decrease in LV-MI was statistically not significant. Also, the variation between LV-CABG and LV-MI (*P* = 0.2368) groups were statistically not significant (Fig. 7A and B). PCR data revealed that both the ISC and ISC/R CF displayed significantly decreased expression of Nrf2 compared to the control (*P* = 0.0034 and *P* < 0.0001 respectively) and the level of Nrf2 transcript was significantly lower in ISC/R compared to the ISC (*P* = 0.0014) CF (Fig. 7C). Nrf2 expression was significantly lower in the ISC CF than the control (*P* = 0.0552) and ISC/R (*P* = 0.0047) groups as revealed by immunostaining and was increased in the ISC/R (*P* = 0.1432) than the control group; however, was statistically not significant (Fig. 7D and E). Western blot showed that the level of Nrf2 was significantly upregulated in the ISC (*P* = 0.0007) CF and was downregulated the in ISC/R (*P* = 0.8611) CF compared to control. Also, the level of Nrf2 was significantly decreased in the ISC/R groups compared to the ISC CF (*P* = 0.0004) (Fig. 7F and G).

#### Nrf2 + Cluster 1 CF represent proinflammatory phenotype

Single cell genomics revealed that 85 CF cells were positive for Nrf2 (FC > 4), which constituted 0.28% of the parent population where 64 cells were mapped in the ISC group (75.29% of Nrf2 + population), and 21 cells in the ISC/R group (24.71% of Nrf2 + population). Overall, the Nrf2 + cells favored the ISC group and were mapped in 3 clusters (Fig. 7H and I). Nrf2 + Cluster 1 displayed 64 cells where 100% cells were mapped in the ISC group based on the distribution of 5817 genes in Nrf2 + Cluster 1 CF based on the expression status in ISC group (Fig. 7J) (Supplementary File 1). ADAMTS1 (FC + 5.19, *P* = 1.0000), S1PR1 (sphingosine-1-phosphate receptor 1) (FC + 4.64, *P* = 0.00539) and AREG (amphiregulin) (FC + 3.94, *P* = 0.0246) were the major upregulated genes; however, the upregulation was statistically not significant for ADAMTS1 (Fig. 7J) (Supplementary File 1). CCL5 (FC –6.66, *P* = 0.1000), CBLN4 (cerebellin 4) (FC –6.29, *P* < 0.0001), and MX2 (FC –6.05, *P* < 0.0001) were the most downregulated genes in the Nrf2 + Cluster 1 cells (Fig. 7J) (Supplementary File 1). Interestingly, most of the upregulated genes in the Nrf2 + Cluster 1 cells were significantly downregulated and vice versa in the Clusters 2 suggesting the contrasting phenotypes (Fig. 7J). The pathway analysis of Nrf2 + Cluster 1 cells displayed the (1) chemokine signaling (Supplementary Fig. 3D), (2) TNF signaling suggesting (Supplementary Fig. 3E), and (3) NLR signaling suggesting a pro-inflammatory phenotype (Supplementary Fig. 3F).

### Ischemia resulted in the decreased Troponin I in the LV tissues and increased levels in the CF

**Fig. 8:**
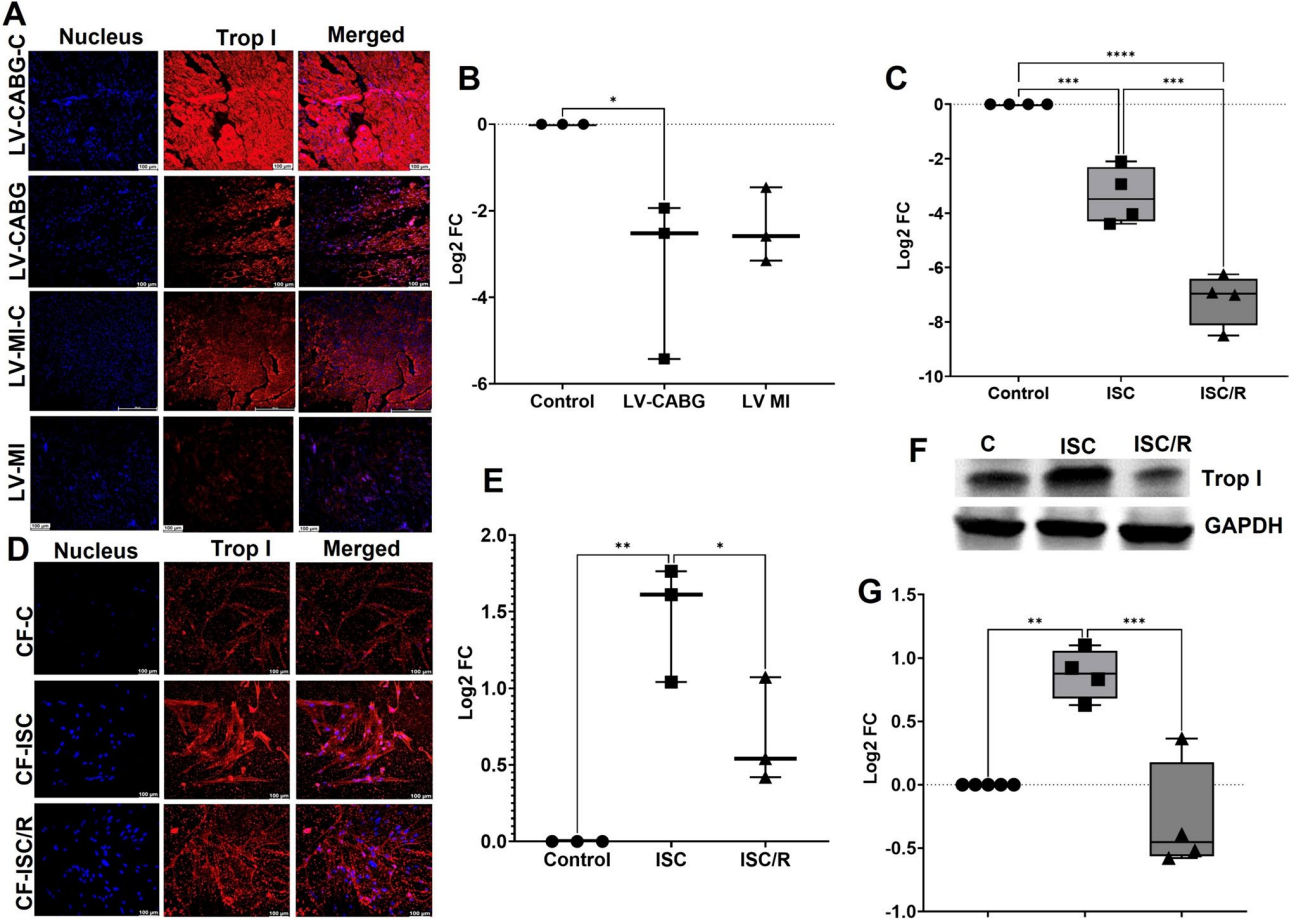
Representative images for the immunofluorescence analysis for the expression of Troponin-I (Trop I) in the LV (*N* = 3) (**A**) tissues and the quantification of protein expression of Troponin-I in LV tissues calculated from MFI (**B**). **C** Quantification of Troponin-I gene expression in CF (*N* = 4) following the treatment with ISC and ISC/R using qRT-PCR. Representative images for the immunofluorescence and quantification for the expression of Troponin-I in the CF (*N* = 3) (**D** and **E**) following the treatment with ISC and ISC/R. **F** Representative image of Western blot and (**G**) quantification showing the expression status of Troponin-I in the ISC and ISC/R groups compared to the control in CF (*N* = 4) (**** P < 0.0001, *** P < 0.001, ** P < 0.01, * P < *0.05, and unlabeled groups represent NS*)

Troponin I was significantly decreased in LV-CABG (*P* = 0.0335); however, the decrease in LV-MI (*P* = 0.1054) was statistically not significant compared to the control. Also, the variation between LV-CABG and LV-MI (*P* = 0.2134) groups were statistically not significant (Fig. 8A and B). Troponin I transcription was significantly downregulated in both ISC (*P* = 0.0006), and ISC/R (*P* < 0.0001) CF compared to the control and the difference between ISC and ISC/R (*P* = 0.0003), were statistically significant (Fig. 8C). Immunostaining revealed that both the ISC (*P* = 0.0022) and ISC/R (*P* = 0.0710) CF displayed increased level of Troponin I; however, the increase in ISC/R CF was statistically not significant compared to the control. Interestingly, the level of Troponin I was significantly lower in the ISC/R group compared to the ISC CF (*P* = 0.0392) (Fig. 8D and E). Western blot analysis revealed a significant upregulation of Troponin I in the ISC (*P* < 0.0001) and a non-significant downregulation in ISC/R (*P* = 0.2932) CF compared to the control. The upregulation of ISC was significantly higher compared to the ISC/R CF (*P* = 0.000) (Fig. 8F and G).

## Discussion

Ischemic myocardial injury results in irreversible loss of cardiomyocytes and the first-line treatment focuses on the rapid restoration of the blood flow (CABG) and to control the hyper-inflammatory responses in the surviving myocardium (Pluijmert et al. 2021) (Ang et al. 2008). Interestingly, the multiphasic functions and interactions of CF, the major cell types in the myocardium second to cardiomyocytes, during the ischemic episodes from proinflammatory to pro-healing phenotypes have been unveiled (Molenaar et al. 2021) (Ko et al. 2022). In addition, our recent report demonstrated the heterogeneity of ischemic stromal fibroblasts harvested from the infarct zone of MI-swine model at single cell resolution unveiled the inflammatory and regenerative phenotypes (Thankam et al. 2023). Also, the plethora of mediators secreted by the CF at the infarct zone coordinate diverse cellular and molecular events associated with myocardial pathology and/or healing suggesting that the secretory components of ischemic stromal fibroblasts are crucial in driving the myocardial pathology/healing (Molenaar et al. 2021). Hence, we aimed to screen the major protein mediators secreted by CF challenged with ischemia and to explore their expression status at the tissue level in the LV harvested from the infarcted and CABG LV tissues of respective swine models and at the cellular level using CF from the ischemic myocardium.

The proteomics analysis revealed a battery of significantly altered mediators secreted by the ischemia challenged CF where Cofilin 1, and HSP90 (exclusively downregulated under ischemia) and CRSP2, HSP27, and IL8 (exclusively upregulated under ischemia) were considered based on their functional role in cardiac tissue and relative abundance. Since oxidative stress and ischemia are intimately related in cardiac pathology, we have explored Nrf2, the master regulator for antioxidant response and Troponin I, the MI biomarker apart from the secreted protein candidates. We have utilized LV from our established models of CABG (Radwan et al. 2022) (Thankam et al. 2020) and MI (Thankam et al. 2022c) to assess the expression status of these mediators in the infarct zone LV tissues. Despite the beneficial outcomes of CABG, the sustenance of ischemic insults in the LV at the vicinity of anastomoses often results in poor outcomes and complications representing a chronic ischemic milieu as in MI (Biancari et al. 2018). The histological examinations revealed increased calcification and fibrosis in the LV tissues compared to the control suggesting the ongoing pathological episodes. Importantly, our previous study established that CF reflect the cardiac pathology and the regenerative communication under ischemia (Thankam et al. 2022d) which encouraged us to further explore the transcription status of the identified mediators in these cells under simulated ischemic conditions.

Cofilin 1 regulates the actin function and is intimately involved in the pro-apoptosis by interfering the mitochondrial integrity (Chua et al. 2003) (Klamt et al. 2009). Hence, the decreased level of Cofilin 1 protein in the LV tissues and the downregulation of Cofilin 1 transcripts and proteins in the CF reflect the improved mitochondrial integrity and the adaptability of the surviving cells to withstand apoptosis. Moreover, the expression status of Cofilin 1 was consistent with the secretory levels as detected by the MS/MS. The Cofilin-1 + Cluster 1 cells displayed an upregulation of ADAMTS5, the protease that degrade versican, a key proteoglycan in cardiac ECM. As versican accumulation is strongly associated with cardiac fibrosis (Barallobre-Barreiro et al. 2021), ADAMTS5 mediated clearance of versican alleviate the cardiac pathology. Hence, Cofilin-1 + Cluster 1 CF suggests the pro-survival phenotype. The upregulation of IGF1 (the major pro-survival mediator in the cardiac tissue) and the downstream mediator IGFBP5 in the Cofilin-1 + Cluster 1 cells supports the pro-survival function (Macvanin et al. 2023) (Guo et al. 2022) (Díaz del Moral et al. 2022). Additionally, the downregulation of cell cycle genes in the Cofilin-1 + Cluster 1 CF reflects the secretory phenotypes (Li et al. 2019) (Ballweg et al. 2018). Hence, Cofilin-1 + Cluster 1 CF especially, Cofilin-1 + ADAMST5 + IGF5 + , represent a protective phenotype; however, warrants further investigations for transcriptional applications.

Cofilin-1 + Cluster 2 cells upregulated HPS5 gene which is involved in the biogenesis of lysosomal organelles complex 2 subunit 2; however, the role of HPS5 in cardiac pathology is largely unknown. MGP has been hailed for the immunomodulatory effects (Feng et al. 2018); however, its carboxylation state is crucial in determining the cardioprotective functions (Malhotra et al. 2022). Similarly, despite a few reports on the immune functions of MX2, its role in cardiac pathology has not been unveiled (Meng et al. 2022). Notably, Cofilin-1 + Cluster 2 cells displayed the downregulation of cell cycle genes suggesting the secretory phenotypes as in Cofilin-1 + Cluster 1 cells. Additionally, the pathway analysis signified the operation of pro-inflammatory pathways in Cofilin-1 + Cluster 2 cells highlighting Cofilin-1 + MGP + MX2 + to be a pro-inflammatory phenotype. On the other hand, Cofilin-1 + Cluster 3 cells displayed a proliferative phenotype, representing the progenitor population (Zhang et al. 2001). Overall, the data suggests that consideration of Cofilin-1 + sub-phenotypes are critical in designing translational experiments.

CRSP2 is involved in calcitonin signaling regulating calcium homeostasis and the information regarding CRSP2 function in ischemic cardiac pathology or healing is largely unknown. However, the calcitonin elicits paracrine signaling from the surviving cardiomyocytes to regulate the fibroblast activation preventing the fibrotic responses indicating cardioprotective function (Davidson et al. 2021). The upregulation of CRSP2 in the CABG myocardial tissues and ischemic CF reflect the activation of calcium signaling following the ischemic insults; however, the exact role of CRSP2 in the ischemic myocardium is unknown. Additionally, CRSP2 + Cluster 1 cells displayed the upregulation of IGFBP5, IGF1, and ADAMTS5 (Barallobre-Barreiro et al. 2021) (Macvanin et al. 2023) (Guo et al. 2022) (Díaz del Moral et al. 2022) as in Cofilin-1 + Cluster 1 cells suggesting the protective phenotype. Moreover, the downregulation of cell cycle mediators reflects the non-proliferative phenotype (Li et al. 2019) (Ballweg et al. 2018). Taken together, the CRSP2 + IGF1 + ADAMTS5 + cells suggest a cardioprotective phenotype.

Extracellular HSP27 exerts protective responses against myocardial ischemia; however, acts as potent DAMP when released into the extracellular space triggering sterile inflammation (Jin et al. 2014) (Hong et al. 2021). Importantly, the cellular overexpression of HSP27 prevents apoptosis, infarction, oxidative damage, contractile dysfunction, and myofilament instability (Lu et al. 2008). The increased level of secretory HSP27 by the ischemic CF observed in our MS/MS data confirms the pro-inflammatory episodes which subsides following the reperfusion. Also, the increased HSP27 expression in the CABG-LV tissues signifies the stable myocardium persisting the chronic ischemic insults compared to the MI-LV tissues which represent an acute ischemic milieu. Interestingly, HSP27 + Cluster 1 cells (favored ischemia) were signatured with the cardioprotective mediators IGF1 and IGFBP5 and ARLD4, where the role of ARLD4 in cardiac pathology/regeneration is largely unknown. The concomitant downregulation of the pathological remodeling mediator ACTA1 (Kern et al. 2014), and the immune mediator MX2 (Meng et al. 2022) along with the operation of pro-survival pathways support the cardioprotective signature of HSP27 + Cluster 1 cells. Interestingly, HSP27 + Cluster 2 cells (favored reperfusion group) displayed exactly contrasting phenotype suggesting the reversal of ischemia compared to the HSP27 + Cluster 1 cells that favored ischemia. Overall, the HSP27 + IGF1 + cells in the Cluster 1 represent a pro-survival phenotype.

IL-8 upregulation and release has been associated with MI which triggers acute inflammation by accelerating the recruitment of neutrophils into the infarct zone (Sun et al. 2019) (Shetelig et al. 2018). The increased level of secretory IL-8 by the ischemic CF from our MS/MS data confirms the aggravated inflammatory signaling. Importantly, the LV-CABG tissues displayed downregulation of IL-8 compared to the respective MI tissues reflecting the pro-inflammatory events under acute ischemia. IL8 + Cluster 1 CF favored reperfusion group characterized by the upregulation of MYBPH, SFRP1, and GLIPR1. Being a key player of contractile apparatus, MYBPH is actively involved in the compensatory mechanisms following ischemia (Mouton et al. 2016). SFRP1 elicits protective effects in cardiac tissue by blocking Wnt signaling and supports cardiac regeneration (Guan et al. 2021) whereas the involvement of the tumor suppressor gene GLIPR1 (Sheng et al. 2016) in cardiac function is currently unknown. The downregulated genes including SLP1, a cardiac inflammatory marker (Sawicki et al. 2023), ECSCR, an apoptotic mediator (Kilari et al. 2013), and EMCN, an inhibitor of endothelial cell integrity (Park-Windhol et al. 2017) were associated with the progression of cardiac pathology. Hence, these observations suggest that IL8 + MYBPH + SFRP1 + Cluster 1 cells represent a cardioprotective phenotype; based on the transcription of signature genes.

HSP90 elicits immense cardioprotective effect by regulating the complement activation (Wang et al. 2020), JNK signaling (Wang et al. 2020) and eNOS phosphorylation (Kupatt et al. 2004) and has been established as a promising therapeutic target for post-MI management (Wang et al. 2020) (Kupatt et al. 2004). The decreased level of secretory HSP90 by the ischemic CF from our MS/MS data confirms the pathological milieu. Contrastingly, increased levels of HSP90 in LV-CABG tissues signify a stable myocardium that withstand chronic ischemic/inflammatory episodes, which was decreased in acute MI. HSP90 + Cluster 1 cells were characterized by the pro-survival mediators including ADAMST5, IGF1, and AKR1C2 with a concomitant downregulation of the inflammatory genes such as SST, CCL5 and MX2. On the other hand, HSP90 + Cluster 2 cells displayed the reverse trend compared to HSP90 + Cluster 1 cells. Hence, HSP90 + ADAMST5 + IGF1 + Cluster 1 cells represent a pro-survival phenotype whereas HSP90 + SST + CCL5 + Cluster 2 cells signify a pro-inflammatory phenotype. Overall, consideration of HSP90 immunopositivity alone is insufficient for designing cell based translational approaches.

Nrf2 induces several cardioprotective genes that play central roles in the defense mechanism against ischemic injury (Zhang et al. 2021) (Mata and Cadenas 2021). Our data revealed the downregulation of Nrf2 in LV tissues and ischemic CF cells suggesting the pro-oxidant environment in the LV and CF reflecting the sustenance of ischemic pathology (Thankam et al. 2020). Nrf2 + Cluster 1 CF favored ischemia and is characterized by the upregulation of S1PR1 (cardioprotective mediator that acts by maintaining calcium homeostasis) (Keul et al. 2016), AREG (pro-fibrotic mediator) (Liu et al. 2020), and ADAMTS1, the ECM protease that supports remodeling (Wang et al. 2021) with a concomitant downregulation of inflammatory mediators MX2 and CCL5. Hence, Nrf2 + S1PR1 + AREG + Cluster 1 CF represent a pro-survival phenotype with translational potential.

### Translational perspective

The findings from this study have revealed several proteins and CF phenotypes with immense translational potential. Based on the trend in upregulation in LV tissues of CABG and MI models, Cofilin 1, CRSP2, IL8, HSP27, HSP90 and CRSP2 play critical roles in chronic and acute ischemic myocardial tissues with the downregulation of Nrf2 and Troponin I. Notably, the upregulation of IGF1, ADAMTS5, and IGFBP5 and concomitant downregulation of CCL5, MX2, and HPS5 in the CF clusters promise cardioprotective populations with therapeutic potential at single cell resolution and vice versa. Further understanding regarding the mechanistic aspects of these phenotypes at cellular and tissue level are warranted for assessing their translational potential. Moreover, these phenotypes are expected to have a significant impact on the ischemic pathology, healing and/or inflammation as these cells are harvested from the ischemic cardiac tissues. However, careful optimization of therapeutic quality phenotypes based on their expression profile and healing mechanisms as these phenotypes are unique with minimal overlapping transcription profile. Hence, further mechanistic, and translational investigations are required to further screen ideal candidates where the possibilities of multiple phenotypes are extremely higher. Additionally, novel flowcytometry based sorting protocols are warranting for sorting and purification of these population for translational applications. Importantly, these findings reveal the future opportunities in the co-administration of multiple regenerative phenotypes for accelerated healing of the myocardium.

To the best of our knowledge this is the first report on the screening while evaluating the biological significance of the major secreted mediators of ischemic CF harvested from translationally relevant swine models. The expression status of Cofilin 1, HSP90, HSP27, IL-8, CRSP2, Troponin 1 and Nrf2 in the LV tissues and CF cells were consistent with the screening profile derived from the MS/MS findings. Importantly, the findings provided novel insights into the biological responses and possible CF phenotypes with regenerative potential; however, detailed mechanistic understanding is warranted. Overall, these proteins play crucial roles in driving the myocardial ischemic pathology/healing machinery exhibiting translational importance in the diagnosis and management of MI. Despite the promising results, our study has limitations including (1) the relative smaller dimension of infarct zone tissues enabled us to rely solely on immunostaining, (2) MS/MS data displayed several mediators which were screened down for further analysis based on abundance in the ISC group; however, other potent mediators may also be possible, (3) the bulk of information from scRNA-seq data resulted in the selection of highly altered genes based on ischemia treatment whereas other targets may be possible, and (4) the cell phenotypes proposed in the study warrants further mechanistic and functional validations. Nonetheless, our findings revealed a panel of protein mediators and unique sub-phenotypes of CF where the further understanding regarding the underlying signaling mechanisms offers novel translational avenues in the management of post-ischemic cardiac complications.

## Conclusions

Based on the mass spectrometry screening three upregulated proteins, CRSP2, HSP27, and IL-8 and two downregulated proteins, Cofilin-1, and HSP90 in the ISC group were critical in the underlying pathophysiology and healing response. Apart from the protein candidates from the MS/MS data, the cardiac biomarker Troponin-I and antioxidant regulator Nrf2 were considered owing to their pathological significance. The expression level of CRSP2, HSP27, IL-8, Cofilin-1, and HSP90 in the LV tissues and CF cells followed the screening profile derived from the MS/MS findings. Single cell genomics revealed multiple sub-phenotypes of CF based on their respective upregulation of CRSP2, HSP27, IL-8, Cofilin-1, HSP90, Troponin I and Nrf2 unveiling pathological and pro-healing phenotypes. Further investigations regarding the underlying signaling mechanisms and validation of sub-populations would offer novel translational avenues for the management of cardiac diseases.

## Supplementary Information

Below is the link to the electronic supplementary material.

Supplementary Figure 1: The PATHVIEW analysis for Cofilin-1+ CF clusters based on the highly altered signature genes using KEGG database: Cofilin-1+ Cluster 1 cells revealing (**A**) calcium signaling, (**B**) HIF1 signaling, and (**C**) p53 signaling; Cofilin-1+ Cluster 2 cells showing (**D**) chemokine signaling, (**E**) TNF signaling, and (**F**) NOD-like receptor signaling; and Cofilin-1+ Cluster 3 cells displaying (**G**) PI3K-AKT signaling and (**H**) cell cycle pathways. (JPG 10558 kb)

Supplementary Figure 2: (**A**) The PATHVIEW analysis for CRSP2+ CF clusters based on the highly altered signature genes using KEGG database showing the activation of cell cycle pathways. The PATHVIEW analysis for HSP27+ CF clusters based on the highly altered signature genes using KEGG database: HSP27+ Cluster 1 cells revealing (**B**) cytokine-cytokine receptor interaction, (**C**) PI3K-AKT signaling, and (**D**) chemokine signaling. The PATHVIEW analysis for IL8+ CF clusters based on the highly altered signature genes using KEGG database: IL8+ Cluster 1 cells revealing (**E**) MAPK signaling, (**F**) cytokine-cytokine receptor interaction, and (**G**) PI3K-AKT signaling. (JPG 13271 kb)

Supplementary Figure 3: The PATHVIEW analysis for HSP90+ CF clusters based on the highly altered signature genes using KEGG database: HSP90+ Cluster 1 cells revealing cytokine-(**A**) cytokine receptor interaction (**B**) NOD-like signaling, and (**C**) TNF pathway. The PATHVIEW analysis for Nrf2+ CF clusters based on the highly altered signature genes using KEGG database: revealing (**D**) chemokine signaling, (**E**) TNF pathway, and (**G**) NOD-like signaling. (JPG 11474 kb)

Supplementary file1 (DOCX 64 kb)

Supplementary file2 (XLSX 2280 kb)

## Data Availability

No datasets were generated or analysed during the current study.
