# Supplementary figures and images for: Ischemic cardiac stromal fibroblast-derived protein mediators in the infarcted myocardium and transcriptomic profiling at single cell resolution
Source: Funct Integr Genomics. 2024 Sep 20;24(5):168. doi: 10.1007/s10142-024-01457-1 (PMC11415418; doi:10.1007/s10142-024-01457-1)

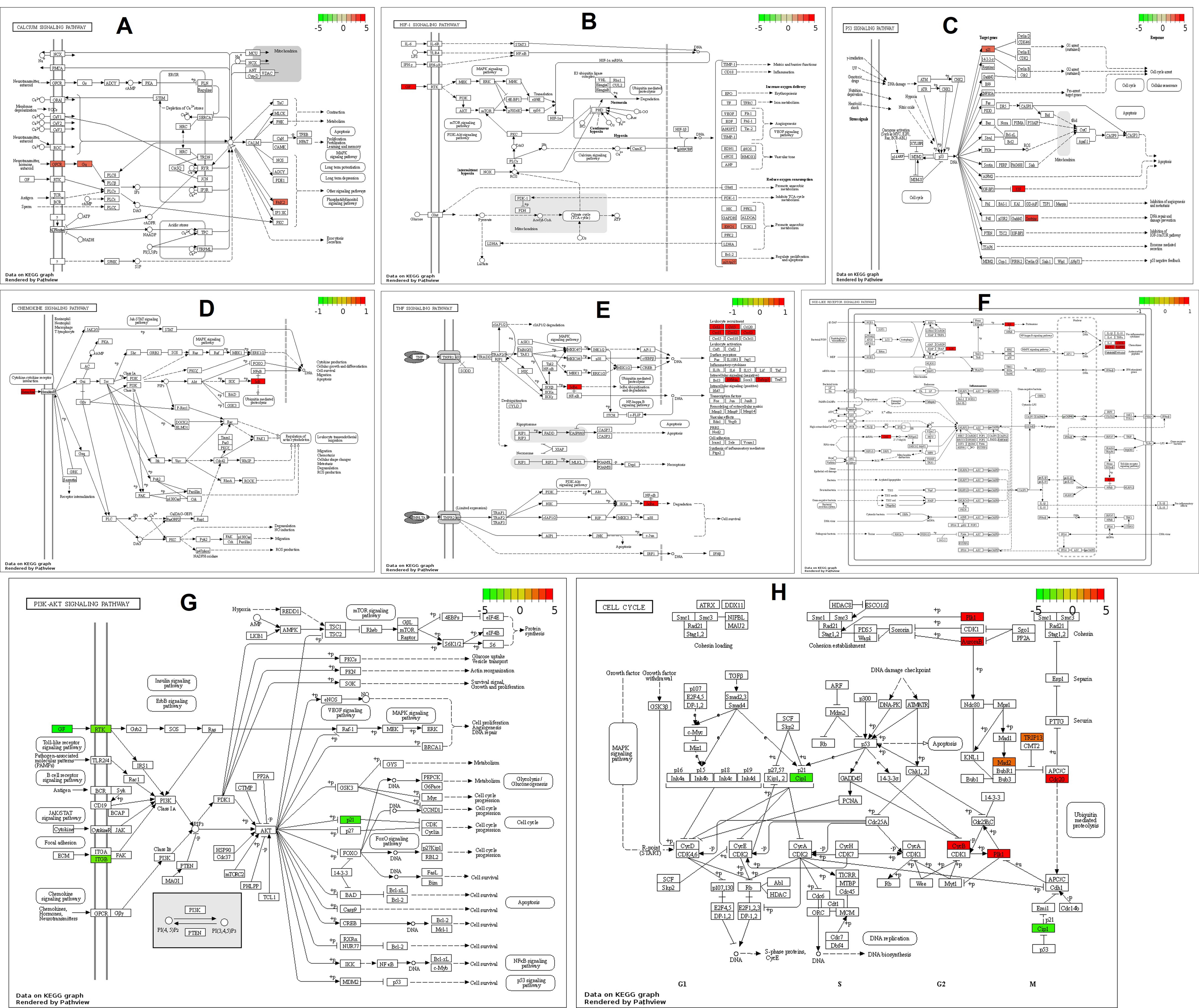

Supplement: Supplementary file 1 — Supplementary Figure 1: The PATHVIEW analysis for Cofilin-1+ CF clusters based on the highly altered signature genes using KEGG database: Cofilin-1+ Cluster 1 cells revealing (A) calcium signaling, (B) HIF1 signaling, and (C) p53 signaling; Cofilin-1+ Cluster 2 cells showing (D) chemokine signaling, (E) TNF signaling, and (F) NOD-like receptor signaling; and Cofilin-1+ Cluster 3 cells displaying (G) PI3K-AKT signaling and (H) cell cycle pathways. (JPG 10558 kb) [file 10142_2024_1457_MOESM1_ESM.jpg]
