# Supplementary material for: Ischemic cardiac stromal fibroblast-derived protein mediators in the infarcted myocardium and transcriptomic profiling at single cell resolution
Source: Funct Integr Genomics. 2024 Sep 20;24(5):168. doi: 10.1007/s10142-024-01457-1 (PMC11415418; doi:10.1007/s10142-024-01457-1)
Supplement: Supplementary file 4 — Supplementary file1 (DOCX 64 kb) [file 10142_2024_1457_MOESM4_ESM.docx]

**Supplementary Table 1**: List and expression status of protein mediators secreted by the CF at ISC and ISC/R.

| **C vs ISC** | | | | | | | | | |
| --- | --- | --- | --- | --- | --- | --- | --- | --- | --- |
| **Sl/No** | **Accession** | **Description** | **Av Log2 FC** | | | | | | **SD** |
| 1 | M3TYT0 | Rho-associated protein kinase 2 | 30.02 | | | | | | 0.0E+00 |
| 2 | P23165 | Protein MGF 360-3L | 28.20 | | | | | | 0.0E+00 |
| 3 | Q766Y7 | Calcitonin receptor-stimulating peptide 2 | 27.98 | | | | | | 4.4E-15 |
| 4 | O46374 | DNA topoisomerase 2-alpha | 26.63 | | | | | | 0.0E+00 |
| 5 | P0CAJ6 | Uncharacterized protein C717R | 26.44 | | | | | | 0.0E+00 |
| 6 | P0C9B0 | Putative ATP-dependent RNA helicase QP509L | 25.99 | | | | | | 0.0E+00 |
| 7 | Q9BDJ5 | Pantetheinase | 25.65 | | | | | | 0.0E+00 |
| 8 | P53366 | Pro-adrenomedullin | 25.64 | | | | | | 3.1E-01 |
| 9 | Q29411 | Chitinase-3-like protein 1 | 25.61 | | | | | | 1.5E-01 |
| 10 | P15468 | Ribonuclease 4 | 25.50 | | | | | | 4.0E-01 |
| 11 | Q28985 | Insulin-like growth factor-binding protein 5 | 25.40 | | | | | | 7.1E-01 |
| 12 | Q5GN48 | Dystrophin | 24.69 | | | | | | 0.0E+00 |
| 13 | Q69DK8 | Complement C1s subcomponent | 24.69 | | | | | | 7.8E-01 |
| 14 | Q65159 | Putative poly(A) polymerase catalytic subunit | 24.36 | | | | | | 4.4E-15 |
| 15 | P79385 | Lactadherin | 24.13 | | | | | | 8.8E-01 |
| 16 | O11780 | Transforming growth factor-beta-induced protein ig-h3 | 23.80 | | | | | | 1.6E+00 |
| 17 | P51779 | Complement factor D | 23.63 | | | | | | 6.6E-01 |
| 18 | Q29030 | Kit ligand | 23.47 | | | | | | 2.4E-01 |
| 19 | Q6SEG5 | Ubiquitin carboxyl-terminal hydrolase isozyme L1 | 23.24 | | | | | | 8.0E-01 |
| 20 | P61288 | Translationally-controlled tumor protein | 23.11 | | | | | | 3.4E-01 |
| 21 | Q03710 | Complement factor B (Fragment) | 22.17 | | | | | | 0.0E+00 |
| 22 | P52552 | Peroxiredoxin-2 (Fragment) | 21.97 | | | | | | 2.0E-01 |
| 23 | Q28944 | Procathepsin L | 21.64 | | | | | | 4.4E-15 |
| 24 | A5A8V7 | Heat shock 70 kDa protein 1-like | 21.46 | | | | | | 0.0E+00 |
| 25 | Q7SIB7 | Phosphoglycerate kinase 1 | 21.46 | | | | | | 0.0E+00 |
| 26 | P0CA10 | Putative helicase/primase complex protein | 21.37 | | | | | | 0.0E+00 |
| 27 | Q28833 | von Willebrand factor (Fragment) | 21.04 | | | | | | 0.0E+00 |
| 28 | Q5S1U1 | Heat shock protein beta-1 | 20.88 | | | | | | 0.0E+00 |
| 29 | O19113 | CCN family member 2 | 20.56 | | | | | | 0.0E+00 |
| 30 | Q29243 | Dystroglycan | 20.39 | | | | | | 0.0E+00 |
| 31 | B3SP85 | Gamma-interferon-inducible-lysosomal thiol reductase | 4.64 | | | | | | 7.1E-01 |
| 32 | P16545 | Insulin-like growth factor I | 4.58 | | | | | | 5.3E-01 |
| 33 | Q95274 | Thymosin beta-4 | 3.79 | | | | | | 3.8E-01 |
| 34 | P79335 | Plasminogen activator inhibitor 1 | 3.65 | | | | | | 4.8E-01 |
| 35 | Q9GKE2 | C-X-C motif chemokine 16 | 3.59 | | | | | | 6.8E-01 |
| 36 | Q9XSD9 | Decorin | 3.33 | | | | | | 2.9E-01 |
| 37 | Q9TV36 | Fibrillin-1 | 3.16 | | | | | | 4.3E-01 |
| 38 | P24854 | Insulin-like growth factor-binding protein 4 | 3.01 | | | | | | 7.4E-01 |
| 39 | P20112 | SPARC | 2.87 | | | | | | 2.8E-01 |
| 40 | Q9GKQ6 | Biglycan (Fragments) | 2.37 | | | | | | 2.6E-01 |
| 41 | P35624 | Metalloproteinase inhibitor 1 | 2.36 | | | | | | 3.9E-01 |
| 42 | P42831 | C-C motif chemokine 2 | 2.13 | | | | | | 5.1E-01 |
| 43 | P22952 | Alveolar macrophage chemotactic factor 2 | 2.01 | | | | | | 9.5E-02 |
| 44 | Q8HZJ6 | Syndecan-4 | 1.98 | | | | | | 6.6E-01 |
| 45 | Q9TTB4 | Fibromodulin (Fragment) | 1.88 | | | | | | 4.2E-01 |
| 46 | P67937 | Tropomyosin alpha-4 chain | 1.70 | | | | | | 2.5E-01 |
| 47 | P42639 | Tropomyosin alpha-1 chain | 1.70 | | | | | | 2.5E-01 |
| 48 | P26042 | Moesin | 1.64 | | | | | | 6.9E-02 |
| 49 | P19620 | Annexin A2 | 1.56 | | | | | | 9.2E-01 |
| 50 | P02543 | Vimentin | 1.52 | | | | | | 1.6E-01 |
| 51 | Q29549 | Clusterin | 1.44 | | | | | | 2.9E-01 |
| 52 | P68137 | Actin, alpha skeletal muscle | 1.43 | | | | | | 1.3E-01 |
| 53 | P00339 | L-lactate dehydrogenase A chain | 1.43 | | | | | | 4.4E-01 |
| 54 | P26234 | Vinculin | 1.41 | | | | | | 4.6E-01 |
| 55 | Q6QAQ1 | Actin, cytoplasmic 1 | 1.38 | | | | | | 1.3E-01 |
| 56 | P00336 | L-lactate dehydrogenase B chain | 1.33 | | | | | | 1.5E-01 |
| 57 | P24853 | Insulin-like growth factor-binding protein 2 | 1.32 | | | | | | 5.8E-01 |
| 58 | Q07717 | Beta-2-microglobulin | 1.24 | | | | | | 1.7E-01 |
| 59 | Q1KYT0 | Beta-enolase | 1.13 | | | | | | 3.3E-01 |
| 60 | Q49I35 | Galectin-1 | 1.12 | | | | | | 1.3E+00 |
| 61 | P62936 | Peptidyl-prolyl cis-trans isomerase A | 1.11 | | | | | | 1.5E-01 |
| 62 | P79379 | Metallothionein-2A | 1.11 | | | | | | 3.0E-01 |
| 63 | P49151 | Vascular endothelial growth factor A | 0.95 | | | | | | 1.4E-16 |
| 64 | Q29371 | Triosephosphate isomerase | 0.92 | | | | | | 3.7E-01 |
| 65 | A1E295 | Cathepsin B | 0.88 | | | | | | 4.9E-01 |
| 66 | P27485 | Retinol-binding protein 4 | 0.71 | | | | | | 4.1E-01 |
| 67 | P20305 | Gelsolin (Fragment) | 0.56 | | | | | | 3.4E-01 |
| 68 | P82460 | Thioredoxin | 0.52 | | | | | | 3.2E-01 |
| 69 | P00761 | Trypsin | 0.39 | | | | | | 8.9E-01 |
| 70 | Q29545 | Inhibitor of carbonic anhydrase | 0.36 | | | | | | 1.4E-01 |
| 71 | Q9GK37 | Corticosteroid-binding globulin | 0.36 | | | | | | 0.0E+00 |
| 72 | P29700 | Alpha-2-HS-glycoprotein (Fragment) | 0.31 | | | | | | 1.2E+00 |
| 73 | P18648 | Apolipoprotein A-I | 0.25 | | | | | | 2.8E-01 |
| 74 | P06867 | Plasminogen | 0.24 | | | | | | 2.0E-01 |
| 75 | O02668 | Inter-alpha-trypsin inhibitor heavy chain H2 | 0.21 | | | | | | 1.5E-01 |
| 76 | P08835 | Albumin | 0.19 | | | | | | 1.7E-01 |
| 77 | P26894 | Interleukin-8 | 0.14 | | | | | | 1.8E-01 |
| 78 | P79263 | Inter-alpha-trypsin inhibitor heavy chain H4 | 0.07 | | | | | | 1.8E-01 |
| 79 | Q06AA3 | Regucalcin | 0.06 | | | | | | 2.8E-02 |
| 80 | P02067 | Hemoglobin subunit beta | -0.03 | | | | | | 2.6E-01 |
| 81 | Q8MJ76 | Alpha-fetoprotein | -0.05 | | | | | | 3.3E-01 |
| 82 | O46409 | Apolipoprotein A-IV | -0.05 | | | | | | 8.5E-18 |
| 83 | P48819 | Vitronectin | -0.08 | | | | | | 2.2E-01 |
| 84 | P00355 | Glyceraldehyde-3-phosphate dehydrogenase | -0.14 | | | | | | 7.1E-01 |
| 85 | P01025 | Complement C3 | -0.20 | | | | | | 5.0E-01 |
| 86 | P49921 | Progonadoliberin-1 | -0.47 | | | | | | 0.0E+00 |
| 87 | P09571 | Serotransferrin | -0.55 | | | | | | 5.6E-01 |
| 88 | Q9TT35 | Thyroxine-binding globulin | -0.55 | | | | | | 4.9E-01 |
| 89 | P04366 | Protein AMBP (Fragment) | -0.77 | | | | | | 0.0E+00 |
| 90 | Q9TUQ3 | Complement component C7 | -0.82 | | | | | | 0.0E+00 |
| 91 | P50447 | Alpha-1-antitrypsin | -0.87 | | | | | | 1.1E+00 |
| 92 | Q767L7 | Tubulin beta chain | -0.94 | | | | | | 0.0E+00 |
| 93 | Q2XVP4 | Tubulin alpha-1B chain | -1.01 | | | | | | 5.5E-01 |
| 94 | P01965 | Hemoglobin subunit alpha | -1.11 | | | | | | 4.7E-01 |
| 95 | P00690 | Pancreatic alpha-amylase | -1.40 | | | | | | 0.0E+00 |
| 96 | Q19AZ8 | Prothrombin | -2.97 | | | | | | 0.0E+00 |
| 97 | P0C9J6 | Protein MGF 360-1L | -20.41 | | | | | | 0.0E+00 |
| 98 | Q95342 | 60S ribosomal protein L18 | -20.57 | | | | | | 0.0E+00 |
| 99 | P61958 | Small ubiquitin-related modifier 2 | -20.75 | | | | | | 0.0E+00 |
| 100 | P60662 | Myosin light polypeptide 6 | -20.75 | | | | | | 0.0E+00 |
| 101 | O02705 | Heat shock protein HSP 90-alpha | -21.22 | | | | | | 0.0E+00 |
| 102 | A3DRP3 | Neuraminidase | -21.98 | | | | | | 4.4E-15 |
| 103 | P10668 | Cofilin-1 | -22.18 | | | | | | 0.0E+00 |
| 104 | Q5G6V9 | Cofilin-2 | -22.18 | | | | | | 0.0E+00 |
| 105 | P62802 | Histone H4 | -22.37 | | | | | | 0.0E+00 |
| 106 | Q3ZD69 | Prelamin-A/C | -22.71 | | | | | | 4.4E-15 |
| 107 | P23695 | Insulin-like growth factor II | -22.93 | | | | | | 0.0E+00 |
| 108 | O02840 | Cadherin-5 | -23.33 | | | | | | 4.4E-15 |
| 109 | Q95242 | Platelet endothelial cell adhesion molecule | -23.55 | | | | | | 0.0E+00 |
| 110 | P79401 | Cytochrome P450 3A29 | -23.93 | | | | | | 0.0E+00 |
| 111 | O97562 | Mitochondrial uncoupling protein 2 | -24.28 | | | | | | 0.0E+00 |
| 112 | A0MD28 | Replicase polyprotein 1ab | -24.62 | | | | | | 0.0E+00 |
| 113 | Q9GMB0 | Dolichyl-diphosphooligosaccharide-protein glycosyltransferase subunit 1 | -25.38 | | | | | | 0.0E+00 |
| 114 | Q5XLD3 | Creatine kinase M-type | -26.79 | | | | | | 0.0E+00 |
| 115 | P16379 | RNA-directed RNA polymerase L | -27.85 | | | | | | 4.4E-15 |
| **C vs ISC/R** | | | | | | | | | |
| **Sl/No** | **Accession** | **Description** | **Av Log2 FC** | | | | | | **SD** |
| 1 | C0HL13 | Low-density lipoprotein receptor-related protein 2 | 28.13 | | | | | | 0.0000 |
| 2 | Q69DK8 | Complement C1s subcomponent | 27.92 | | 0.3282 | | | | |
| 3 | O97763 | NPC intracellular cholesterol transporter 2 | 26.63 | | 0.3523 | | | | |
| 4 | P15468 | Ribonuclease 4 | 26.48 | | 0.2317 | | | | |
| 5 | Q29411 | Chitinase-3-like protein 1 | 26.35 | | 0.7109 | | | | |
| 6 | P20460 | Eukaryotic translation initiation factor 2 subunit 1 (Fragment) | 26.06 | | 0.0000 | | | | |
| 7 | Q29243 | Dystroglycan | 25.58 | | 0.3460 | | | | |
| 8 | O46427 | Pro-cathepsin H | 25.43 | | 0.1969 | | | | |
| 9 | P53366 | Pro-adrenomedullin | 25.18 | | 0.5673 | | | | |
| 10 | O19113 | CCN family member 2 | 25.15 | | 0.2766 | | | | |
| 11 | O11780 | Transforming growth factor-beta-induced protein ig-h3 | 25.04 | | 0.5286 | | | | |
| 12 | P61288 | Translationally-controlled tumor protein | 24.98 | | 0.4199 | | | | |
| 13 | Q2EN76 | Nucleoside diphosphate kinase B | 24.96 | | 1.6312 | | | | |
| 14 | P28491 | Calreticulin | 24.72 | | 0.0000 | | | | |
| 15 | P51779 | Complement factor D | 24.65 | | 0.3710 | | | | |
| 16 | P79385 | Lactadherin | 24.57 | | 0.7805 | | | | |
| 17 | A5A8V7 | Heat shock 70 kDa protein 1-like | 24.45 | | 0.5147 | | | | |
| 18 | Q28944 | Procathepsin L | 24.43 | | 0.2252 | | | | |
| 19 | P0C9F7 | Protein MGF 100-3L | 24.42 | | 0.0000 | | | | |
| 20 | Q29315 | 60S acidic ribosomal protein P2 | 24.38 | | 0.3771 | | | | |
| 21 | Q9BDJ5 | Pantetheinase | 24.36 | | 0.0000 | | | | |
| 22 | Q7M329 | Ribonuclease T2 | 24.02 | | 0.5482 | | | | |
| 23 | F1RRV3 | Thyroglobulin | 24.01 | | 0.0000 | | | | |
| 24 | P52552 | Peroxiredoxin-2 (Fragment) | 23.98 | | 0.7924 | | | | |
| 25 | P08059 | Glucose-6-phosphate isomerase | 23.84 | | 0.1360 | | | | |
| 26 | A0A1S6M251 | Beta-1,4-galactosyltransferase 5 | 23.81 | | 0.3838 | | | | |
| 27 | Q7SIB7 | Phosphoglycerate kinase 1 | 23.77 | | 0.3437 | | | | |
| 28 | Q8QPG7 | Polymerase basic protein 2 | 23.74 | | 0.0000 | | | | |
| 29 | Q28985 | Insulin-like growth factor-binding protein 5 | 23.71 | | 1.4260 | | | | |
| 30 | Q767L6 | Flotillin-1 | 23.59 | | 0.0000 | | | | |
| 31 | Q6SEG5 | Ubiquitin carboxyl-terminal hydrolase isozyme L1 | 23.51 | | 0.0000 | | | | |
| 32 | Q6Q7J2 | Rab GDP dissociation inhibitor beta | 23.44 | | 0.6418 | | | | |
| 33 | P12309 | Glutaredoxin-1 | 23.44 | 0.3148 | | | | | |
| 34 | P0CG68 | Polyubiquitin-C | 23.42 | 0.6177 | | | | | |
| 35 | P81405 | Saposin-B-Val | 23.39 | 0.0000 | | | | | |
| 36 | P45846 | Dermatopontin | 23.24 | 1.3998 | | | | | |
| 37 | P04163 | Protein S100-A10 | 23.20 | 0.5839 | | | | | |
| 38 | Q2TNK5 | Angiopoietin-related protein 4 | 22.83 | 0.4094 | | | | | |
| 39 | B8Y466 | SRSF protein kinase 3 | 22.51 | 0.0000 | | | | | |
| 40 | Q9GLP1 | Coagulation factor V | 22.48 | 0.8620 | | | | | |
| 41 | Q29387 | Elongation factor 1-gamma (Fragment) | 22.38 | 0.7268 | | | | | |
| 42 | P79307 | Amyloid-beta A4 protein | 22.30 | 1.2306 | | | | | |
| 43 | P11708 | Malate dehydrogenase, cytoplasmic | 22.07 | 0.5333 | | | | | |
| 44 | A2BD09 | Olfactomedin-like protein 2A | 22.00 | 0.0000 | | | | | |
| 45 | P29269 | Myosin regulatory light polypeptide 9 | 21.67 | 0.5286 | | | | | |
| 46 | P00795 | Cathepsin D | 21.51 | 0.0000 | | | | | |
| 47 | Q997F1 | Protein C | 21.39 | 0.0000 | | | | | |
| 48 | Q7M2W6 | Alpha-crystallin B chain | 21.37 | 0.0000 | | | | | |
| 49 | P79403 | Neutral alpha-glucosidase AB | 21.05 | 0.0000 | | | | | |
| 50 | P53027 | 60S ribosomal protein L10a (Fragment) | 20.88 | 0.0000 | | | | | |
| 51 | P04088 | Inhibin beta B chain | 20.59 | 0.0000 | | | | | |
| 52 | B5SNZ6 | Actin-binding Rho-activating protein | 19.57 | 0.0000 | | | | | |
| 53 | P80031 | Glutathione S-transferase P | 19.48 | 0.0000 | | | | | |
| 54 | P79335 | Plasminogen activator inhibitor 1 | 5.11 | 0.5433 | | | | | |
| 55 | B3SP85 | Gamma-interferon-inducible-lysosomal thiol reductase | 5.08 | 0.4774 | | | | | |
| 56 | P16545 | Insulin-like growth factor I | 4.57 | 0.2098 | | | | | |
| 57 | Q9XSD9 | Decorin | 4.49 | 0.1933 | | | | | |
| 58 | Q9TV36 | Fibrillin-1 | 4.23 | 0.3830 | | | | | |
| 59 | P60662 | Myosin light polypeptide 6 | 3.77 | 0.1571 | | | | | |
| 60 | Q9GKE2 | C-X-C motif chemokine 16 | 3.67 | 0.6257 | | | | | |
| 61 | Q9GKQ6 | Biglycan (Fragments) | 3.63 | 0.2063 | | | | | |
| 62 | Q95274 | Thymosin beta-4 | 3.58 | 0.4400 | | | | | |
| 63 | P20112 | SPARC | 3.35 | 0.5082 | | | | | |
| 64 | Q07717 | Beta-2-microglobulin | 3.25 | | | | | 0.4385 | |
| 65 | P35624 | Metalloproteinase inhibitor 1 | 3.20 | | | | | 0.5637 | |
| 66 | P24854 | Insulin-like growth factor-binding protein 4 | 3.17 | | | | | 0.5579 | |
| 67 | P26042 | Moesin | 3.11 | | | | | 0.2442 | |
| 68 | Q9TTB4 | Fibromodulin (Fragment) | 3.09 | | | | | 0.1470 | |
| 69 | O02705 | Heat shock protein HSP 90-alpha | 2.89 | | | | | 0.7018 | |
| 70 | Q29549 | Clusterin | 2.87 | | | | | 0.2027 | |
| 71 | P10668 | Cofilin-1 | 2.80 | | | | | 0.2669 | |
| 72 | Q49I35 | Galectin-1 | 2.73 | | | | | 0.4150 | |
| 73 | P00339 | L-lactate dehydrogenase A chain | 2.73 | | | | | 0.4843 | |
| 74 | Q5G6V9 | Cofilin-2 | 2.70 | | | | | 0.0056 | |
| 75 | P42831 | C-C motif chemokine 2 | 2.53 | | | | | 0.0840 | |
| 76 | Q8HZJ6 | Syndecan-4 | 2.35 | | | | | 0.6997 | |
| 77 | P61958 | Small ubiquitin-related modifier 2 | 1.91 | | | | | 0.0377 | |
| 78 | P04366 | Protein AMBP (Fragment) | 1.91 | | | | | 1.2271 | |
| 79 | A1E295 | Cathepsin B | 1.90 | | | | | 0.3891 | |
| 80 | P24853 | Insulin-like growth factor-binding protein 2 | 1.85 | | | | | 0.2072 | |
| 81 | P26234 | Vinculin | 1.82 | | | | | 0.4753 | |
| 82 | P02543 | Vimentin | 1.75 | | | | | 0.2010 | |
| 83 | Q1KYT0 | Beta-enolase | 1.70 | | | | | 0.2491 | |
| 84 | Q6QAQ1 | Actin, cytoplasmic 1 | 1.46 | | | | | 0.2876 | |
| 85 | P20305 | Gelsolin (Fragment) | 1.46 | | | | | 0.3158 | |
| 86 | P00336 | L-lactate dehydrogenase B chain | 1.45 | | | | | 0.1170 | |
| 87 | P67937 | Tropomyosin alpha-4 chain | 1.35 | | | | | 0.0400 | |
| 88 | P68137 | Actin, alpha skeletal muscle | 1.33 | | | | | 0.2885 | |
| 89 | P49921 | Progonadoliberin-1 | 1.30 | | | | | 0.5733 | |
| 90 | P42639 | Tropomyosin alpha-1 chain | 1.23 | | | | | 0.1717 | |
| 91 | P82460 | Thioredoxin | 1.17 | | | | | 0.6318 | |
| 92 | Q29371 | Triosephosphate isomerase | 1.13 | | | | | 0.9118 | |
| 93 | P62936 | Peptidyl-prolyl cis-trans isomerase A | 0.88 | | | | | 0.2634 | |
| 94 | Q9GK37 | Corticosteroid-binding globulin | 0.77 | | | | | 0.0000 | |
| 95 | P22952 | Alveolar macrophage chemotactic factor 2 | 0.70 | | | | 0.3297 | | |
| 96 | P19620 | Annexin A2 | 0.64 | | | | 0.6288 | | |
| 97 | P09571 | Serotransferrin | 0.50 | | | | 0.3959 | | |
| 98 | Q3ZD69 | Prelamin-A/C | 0.43 | | | | 1.5737 | | |
| 99 | Q19AZ8 | Prothrombin | 0.43 | | | | 0.1987 | | |
| 100 | Q9TUQ3 | Complement component C7 | 0.41 | | | | 0.1405 | | |
| 101 | Q9TT35 | Thyroxine-binding globulin | 0.41 | | | | 0.5875 | | |
| 102 | Q2XVP4 | Tubulin alpha-1B chain | 0.35 | | | | 1.0372 | | |
| 103 | P01025 | Complement C3 | 0.35 | | | | 0.4599 | | |
| 104 | P48819 | Vitronectin | 0.19 | | | | 0.0983 | | |
| 105 | Q767L7 | Tubulin beta chain | 0.08 | | | | 0.6624 | | |
| 106 | P79263 | Inter-alpha-trypsin inhibitor heavy chain H4 | 0.06 | | | | 0.7659 | | |
| 107 | P50447 | Alpha-1-antitrypsin | 0.05 | | | | 0.4988 | | |
| 108 | P18648 | Apolipoprotein A-I | 0.03 | | | | 0.3451 | | |
| 109 | P00761 | Trypsin | 0.02 | | | | 0.8488 | | |
| 110 | P02067 | Hemoglobin subunit beta | -0.05 | | | | 0.3696 | | |
| 111 | P00690 | Pancreatic alpha-amylase | -0.10 | | | | 0.1366 | | |
| 112 | P08835 | Albumin | -0.15 | | | | 0.1716 | | |
| 113 | P27485 | Retinol-binding protein 4 | -0.32 | | | | 1.5598 | | |
| 114 | P79379 | Metallothionein-2A | -0.42 | | | | 0.8034 | | |
| 115 | O02668 | Inter-alpha-trypsin inhibitor heavy chain H2 | -0.63 | | | | 0.5484 | | |
| 116 | Q8MJ76 | Alpha-fetoprotein | -0.91 | | | | 1.2707 | | |
| 117 | Q29545 | Inhibitor of carbonic anhydrase | -0.92 | | | | 1.1578 | | |
| 118 | P06867 | Plasminogen | -0.99 | | | | 0.3767 | | |
| 119 | P01965 | Hemoglobin subunit alpha | -0.99 | | | | 0.3030 | | |
| 120 | P00355 | Glyceraldehyde-3-phosphate dehydrogenase | -1.05 | | | | 0.6856 | | |
| 121 | P23695 | Insulin-like growth factor II | -1.79 | | | | 0.0000 | | |
| 122 | Q06AA3 | Regucalcin | -2.43 | | | | 0.0000 | | |
| 123 | P29700 | Alpha-2-HS-glycoprotein (Fragment) | -2.44 | | | | 0.4015 | | |
| 124 | O46409 | Apolipoprotein A-IV | -2.55 | | | | 0.0000 | | |
| 125 | P0C9J6 | Protein MGF 360-1L | -20.41 | | | | 0.0000 | | |
| 126 | Q95342 | 60S ribosomal protein L18 | -20.57 | | | 0.0000 | | | |
| 127 | A3DRP3 | Neuraminidase | -21.98 | | | 0.0000 | | | |
| 128 | P49151 | Vascular endothelial growth factor A | -22.29 | | | 0.0000 | | | |
| 129 | P62802 | Histone H4 | -22.37 | | | 0.0000 | | | |
| 130 | O02840 | Cadherin-5 | -23.33 | | | 0.0000 | | | |
| 131 | Q95242 | Platelet endothelial cell adhesion molecule | -23.55 | | | 0.0000 | | | |
| 132 | P79401 | Cytochrome P450 3A29 | -23.93 | | | 0.0000 | | | |
| 133 | O97562 | Mitochondrial uncoupling protein 2 | -24.28 | | | 0.0000 | | | |
| 134 | A0MD28 | Replicase polyprotein 1ab | -24.62 | | | 0.0000 | | | |
| 135 | Q9GMB0 | Dolichyl-diphosphooligosaccharide--protein glycosyltransferase subunit 1 | -25.38 | | | 0.0000 | | | |
| 136 | P26894 | Interleukin-8 | -26.31 | | | 0.0000 | | | |
| 137 | Q5XLD3 | Creatine kinase M-type | -26.79 | | | 0.0000 | | | |
| 138 | P16379 | RNA-directed RNA polymerase L | -27.85 | | | 0.0000 | | | |
| **ISC vs ISC/R** | | | | | | | | | |
| **Sl/No** | **Accession** | **Description** | **Log2 FC** | | | | | | **SD** |
| 1 | C0HL13 | Low-density lipoprotein receptor-related protein 2 | 28.12505 | | | | | | 0.0000 |
| 2 | O97763 | NPC intracellular cholesterol transporter 2 | 26.62557 | | | | | | 0.3523 |
| 3 | P20460 | Eukaryotic translation initiation factor 2 subunit 1 (Fragment) | 26.05591 | | | | | | 0.0000 |
| 4 | O46427 | Pro-cathepsin H | 25.43467 | | | | | | 0.1969 |
| 5 | P10668 | Cofilin-1 | 24.98137 | | | | | | 0.2669 |
| 6 | Q2EN76 | Nucleoside diphosphate kinase B | 24.9633 | | | | | | 1.6312 |
| 7 | Q5G6V9 | Cofilin-2 | 24.88385 | | | | | | 0.0056 |
| 8 | P60662 | Myosin light polypeptide 6 | 24.52369 | | | | | | 0.1571 |
| 9 | P0C9F7 | Protein MGF 100-3L | 24.42306 | | | | | | 0.0000 |
| 10 | Q29315 | 60S acidic ribosomal protein P2 | 24.385 | | | | | | 0.3771 |
| 11 | O02705 | Heat shock protein HSP 90-alpha | 24.10347 | | | | | | 0.7018 |
| 12 | Q7M329 | Ribonuclease T2 | 24.02452 | | | | | | 0.5482 |
| 13 | F1RRV3 | Thyroglobulin | 24.01151 | | | | | | 0.0000 |
| 14 | P08059 | Glucose-6-phosphate isomerase | 23.84429 | | | | | | 0.1360 |
| 15 | A0A1S6M251 | Beta-1,4-galactosyltransferase 5 | 23.81019 | | | | | | 0.3838 |
| 16 | Q8QPG7 | Polymerase basic protein 2 | 23.73658 | | | | | | 0.0000 |
| 17 | Q767L6 | Flotillin-1 | 23.59108 | | | | | | 0.0000 |
| 18 | Q6Q7J2 | Rab GDP dissociation inhibitor beta | 23.44474 | | | | | | 0.6418 |
| 19 | P12309 | Glutaredoxin-1 | 23.44098 | | | | | | 0.3148 |
| 20 | P0CG68 | Polyubiquitin-C | 23.41802 | | | | | | 0.6177 |
| 21 | P81405 | Saposin-B-Val | 23.38601 | | | | | | 0.0000 |
| 22 | P45846 | Dermatopontin | 23.23759 | | | | | | 1.3998 |
| 23 | P04163 | Protein S100-A10 | 23.20497 | | | | | | 0.5839 |
| 24 | Q3ZD69 | Prelamin-A/C | 23.14479 | | | | | | 1.5737 |
| 25 | P28491 | Calreticulin | 23.1313 | | | | | | 0.0000 |
| 26 | Q2TNK5 | Angiopoietin-related protein 4 | 22.83224 | | | | | | 0.4094 |
| 27 | P61958 | Small ubiquitin-related modifier 2 | 22.6568 | | | | | | 0.0377 |
| 28 | B8Y466 | SRSF protein kinase 3 | 22.50846 | | | | | | 0.0000 |
| 29 | Q9GLP1 | Coagulation factor V | 22.48199 | | | | | | 0.8620 |
| 30 | Q29387 | Elongation factor 1-gamma (Fragment) | 22.38132 | | | | | | 0.7268 |
| 31 | P79307 | Amyloid-beta A4 protein | 22.29838 | | | | | | 1.2306 |
| 32 | P11708 | Malate dehydrogenase, cytoplasmic | 22.07083 | | | | | | 0.5333 |
| 33 | A2BD09 | Olfactomedin-like protein 2A | 22.0009 | | | | | | 0.0000 |
| 34 | P29269 | Myosin regulatory light polypeptide 9 | 21.67181 | | | | | | 0.5286 |
| 35 | P00795 | Cathepsin D | 21.50545 | | | | | | 0.0000 |
| 36 | Q997F1 | Protein C | 21.394 | | | | | | 0.0000 |
| 37 | Q7M2W6 | Alpha-crystallin B chain | 21.36526 | | | | | | 0.0000 |
| 38 | P23695 | Insulin-like growth factor II | 21.1431 | | | | | | 0.0000 |
| 39 | P79403 | Neutral alpha-glucosidase AB | 21.04965 | | | | | | 0.0000 |
| 40 | P53027 | 60S ribosomal protein L10a (Fragment) | 20.88117 | | | | | | 0.0000 |
| 41 | P04088 | Inhibin beta B chain | 20.58606 | | | | | | 0.0000 |
| 42 | B5SNZ6 | Actin-binding Rho-activating protein | 19.57275 | | | | | | 0.0000 |
| 43 | P80031 | Glutathione S-transferase P | 19.47755 | | | | | | 0.0000 |
| 44 | Q29243 | Dystroglycan | 5.184654 | | | | | | 0.3460 |
| 45 | O19113 | CCN family member 2 | 4.593323 | | | | | | 0.2766 |
| 46 | Q19AZ8 | Prothrombin | 3.397527 | | | | | | 0.1987 |
| 47 | Q69DK8 | Complement C1s subcomponent | 3.087988 | | | | | | 0.3282 |
| 48 | A5A8V7 | Heat shock 70 kDa protein 1-like | 2.988423 | | | | | | 0.5147 |
| 49 | Q28944 | Procathepsin L | 2.790159 | | | | | | 0.2252 |
| 50 | P04366 | Protein AMBP (Fragment) | 2.678988 | | | | | | 1.2271 |
| 51 | P52552 | Peroxiredoxin-2 (Fragment) | 2.590208 | | | | | | 0.7924 |
| 52 | P61288 | Translationally-controlled tumor protein | 2.432841 | | | | | | 0.4199 |
| 53 | Q7SIB7 | Phosphoglycerate kinase 1 | 2.315273 | | | | | | 0.3437 |
| 54 | P26042 | Moesin | 2.049912 | | | | | | 0.2442 |
| 55 | Q07717 | Beta-2-microglobulin | 2.003447 | | | | | | 0.4385 |
| 56 | P00339 | L-lactate dehydrogenase A chain | 1.854711 | | | | | | 0.4843 |
| 57 | P49921 | Progonadoliberin-1 | 1.774519 | | | | | | 0.5733 |
| 58 | Q9TV36 | Fibrillin-1 | 1.622613 | | | | | | 0.3830 |
| 59 | P15468 | Ribonuclease 4 | 1.533959 | | | | | | 0.2317 |
| 60 | Q29549 | Clusterin | 1.415806 | | | | | | 0.2027 |
| 61 | P79335 | Plasminogen activator inhibitor 1 | 1.404108 | | | | | | 0.5433 |
| 62 | Q49I35 | Galectin-1 | 1.308937 | | | | | | 0.4150 |
| 63 | P00690 | Pancreatic alpha-amylase | 1.306192 | | | | | | 0.1366 |
| 64 | Q2XVP4 | Tubulin alpha-1B chain | 1.297513 | | | | | | 1.0372 |
| 65 | Q9GKQ6 | Biglycan (Fragments) | 1.250009 | | | | | | 0.2063 |
| 66 | Q9TUQ3 | Complement component C7 | 1.236421 | | | | | | 0.1405 |
| 67 | Q9TTB4 | Fibromodulin (Fragment) | 1.168498 | | | | | | 0.1470 |
| 68 | Q9XSD9 | Decorin | 1.134368 | | | | | | 0.1933 |
| 69 | Q767L7 | Tubulin beta chain | 1.017548 | | | | | | 0.6624 |
| 70 | P09571 | Serotransferrin | 0.982544 | | | | | | 0.3959 |
| 71 | A1E295 | Cathepsin B | 0.973057 | | | | | | 0.3891 |
| 72 | B3SP85 | Gamma-interferon-inducible-lysosomal thiol reductase | 0.935894 | | | | | | 0.4774 |
| 73 | P51779 | Complement factor D | 0.921112 | | | | | | 0.3710 |
| 74 | Q9TT35 | Thyroxine-binding globulin | 0.910817 | | | | | | 0.5875 |
| 75 | P20305 | Gelsolin (Fragment) | 0.866892 | | | | | | 0.3158 |
| 76 | P35624 | Metalloproteinase inhibitor 1 | 0.79649 | | | | | | 0.5637 |
| 77 | Q6SEG5 | Ubiquitin carboxyl-terminal hydrolase isozyme L1 | 0.755192 | | | | | | 0.0000 |
| 78 | Q29411 | Chitinase-3-like protein 1 | 0.731644 | | | | | | 0.7109 |
| 79 | O11780 | Transforming growth factor-beta-induced protein ig-h3 | 0.705562 | | | | | | 0.5286 |
| 80 | P00336 | L-lactate dehydrogenase B chain | 0.705471 | | | | | | 0.1170 |
| 81 | P82460 | Thioredoxin | 0.626012 | | | | | | 0.6318 |
| 82 | P50447 | Alpha-1-antitrypsin | 0.618415 | | | | | | 0.4988 |
| 83 | Q9GKE2 | C-X-C motif chemokine 16 | 0.591778 | | | | | | 0.6257 |
| 84 | Q1KYT0 | Beta-enolase | 0.543155 | | | | | | 0.2491 |
| 85 | P01025 | Complement C3 | 0.492334 | | | | | | 0.4599 |
| 86 | P20112 | SPARC | 0.464606 | | | | | | 0.5082 |
| 87 | P24853 | Insulin-like growth factor-binding protein 2 | 0.458361 | | | | | | 0.2072 |
| 88 | Q9GK37 | Corticosteroid-binding globulin | 0.41105 | | | | | | 0.0000 |
| 89 | P26234 | Vinculin | 0.366049 | | | | | | 0.4753 |
| 90 | P42831 | C-C motif chemokine 2 | 0.339751 | | | | | | 0.0840 |
| 91 | P79385 | Lactadherin | 0.282124 | | | | | | 0.7805 |
| 92 | Q8HZJ6 | Syndecan-4 | 0.268434 | | | | | | 0.6997 |
| 93 | P48819 | Vitronectin | 0.253322 | | | | | | 0.0983 |
| 94 | P02543 | Vimentin | 0.222429 | | | | | | 0.2010 |
| 95 | Q29371 | Triosephosphate isomerase | 0.177358 | | | | | | 0.9118 |
| 96 | P53366 | Pro-adrenomedullin | 0.108921 | | | | | | 0.5673 |
| 97 | Q6QAQ1 | Actin, cytoplasmic 1 | 0.075787 | | | | | | 0.2876 |
| 98 | P01965 | Hemoglobin subunit alpha | 0.070136 | | | | | | 0.3030 |
| 99 | P24854 | Insulin-like growth factor-binding protein 4 | 0.021849 | | | | | | 0.5579 |
| 100 | P79263 | Inter-alpha-trypsin inhibitor heavy chain H4 | -0.0155 | | | | | | 0.7659 |
| 101 | P02067 | Hemoglobin subunit beta | -0.03345 | | | | | | 0.3696 |
| 102 | P16545 | Insulin-like growth factor I | -0.07089 | | | | | | 0.2098 |
| 103 | P68137 | Actin, alpha skeletal muscle | -0.11227 | | | | | | 0.2885 |
| 104 | P62936 | Peptidyl-prolyl cis-trans isomerase A | -0.23765 | | | | | | 0.2634 |
| 105 | P18648 | Apolipoprotein A-I | -0.24278 | | | | | | 0.3451 |
| 106 | Q95274 | Thymosin beta-4 | -0.2518 | | | | | | 0.4400 |
| 107 | P08835 | Albumin | -0.34763 | | | | | | 0.1716 |
| 108 | P67937 | Tropomyosin alpha-4 chain | -0.35888 | | | | | | 0.0400 |
| 109 | P42639 | Tropomyosin alpha-1 chain | -0.4822 | | | | | | 0.1717 |
| 110 | P00761 | Trypsin | -0.53242 | | | | | | 0.8488 |
| 111 | O02668 | Inter-alpha-trypsin inhibitor heavy chain H2 | -0.84182 | | | | | | 0.5484 |
| 112 | Q8MJ76 | Alpha-fetoprotein | -0.88088 | | | | | | 1.2707 |
| 113 | P00355 | Glyceraldehyde-3-phosphate dehydrogenase | -1.02063 | | | | | | 0.6856 |
| 114 | P27485 | Retinol-binding protein 4 | -1.06884 | | | | | | 1.5598 |
| 115 | P19620 | Annexin A2 | -1.08818 | | | | | | 0.6288 |
| 116 | P06867 | Plasminogen | -1.23233 | | | | | | 0.3767 |
| 117 | Q29545 | Inhibitor of carbonic anhydrase | -1.28461 | | | | | | 1.1578 |
| 118 | Q9BDJ5 | Pantetheinase | -1.2919 | | | | | | 0.0000 |
| 119 | P22952 | Alveolar macrophage chemotactic factor 2 | -1.3073 | | | | | | 0.3297 |
| 120 | P79379 | Metallothionein-2A | -1.55275 | | | | | | 0.8034 |
| 121 | Q28985 | Insulin-like growth factor-binding protein 5 | -1.80163 | | | | | | 1.4260 |
| 122 | Q06AA3 | Regucalcin | -1.89928 | | | | | | 0.0000 |
| 123 | O46409 | Apolipoprotein A-IV | -2.49589 | | | | | | 0.0000 |
| 124 | P29700 | Alpha-2-HS-glycoprotein (Fragment) | -3.02036 | | | | | | 0.4015 |
| 125 | Q5S1U1 | Heat shock protein beta-1 | -20.8822 | | | | | | 0.0000 |
| 126 | Q28833 | von Willebrand factor (Fragment) | -21.0387 | | | | | | 0.0000 |
| 127 | P0CA10 | Putative helicase/primase complex protein | -21.365 | | | | | | 0.0000 |
| 128 | Q03710 | Complement factor B (Fragment) | -22.1654 | | | | | | 0.0000 |
| 129 | P49151 | Vascular endothelial growth factor A | -23.246 | | | | | | 0.0000 |
| 130 | Q29030 | Kit ligand OS=Sus scrofa | -23.4844 | | | | | | 0.0000 |
| 131 | Q65159 | Putative poly(A) polymerase catalytic subunit | -24.3635 | | | | | | 0.0000 |
| 132 | Q5GN48 | Dystrophin | -24.6872 | | | | | | 0.0000 |
| 133 | P0C9B0 | Putative ATP-dependent RNA helicase QP509L | -25.9876 | | | | | | 0.0000 |
| 134 | P0CAJ6 | Uncharacterized protein C717R | -26.4359 | | | | | | 0.0000 |
| 135 | P26894 | Interleukin-8 | -26.4526 | | | | | | 0.0000 |
| 136 | O46374 | DNA topoisomerase 2-alpha | -26.6279 | | | | | | 0.0000 |
| 137 | Q766Y7 | Calcitonin receptor-stimulating peptide 2 | -27.9847 | | | | | | 0.0000 |
| 138 | P23165 | Protein MGF 360-3L | -28.2001 | | | | | | 0.0000 |
| 139 | M3TYT0 | Rho-associated protein kinase 2 | -30.0234 | | | | | | 0.0000 |
